# Bayesian DNA copy number analysis

**DOI:** 10.1186/1471-2105-10-10

**Published:** 2009-01-08

**Authors:** Paola MV Rancoita, Marcus Hutter, Francesco Bertoni, Ivo Kwee

**Affiliations:** 1 Istituto Dalle Molle di Studi sull'Intelligenza Artificiale (IDSIA), Galleria 2, 6928 Manno-Lugano, Switzerland; 2 Laboratory of Experimental Oncology, Oncology Institute of Southern Switzerland (IOSI), via Vela 6, 6500 Bellinzona, Switzerland; 3 Dipartimento di Matematica, Università degli Studi di Milano, via Saldini 50, 20137 Milano, Italy; 4 RSISE, ANU and SML, NICTA, Canberra, ACT, 0200, Australia

## Abstract

**Background:**

Some diseases, like tumors, can be related to chromosomal aberrations, leading to changes of DNA copy number. The copy number of an aberrant genome can be represented as a piecewise constant function, since it can exhibit regions of deletions or gains. Instead, in a healthy cell the copy number is two because we inherit one copy of each chromosome from each our parents.

Bayesian Piecewise Constant Regression (BPCR) is a Bayesian regression method for data that are noisy observations of a piecewise constant function. The method estimates the unknown segment number, the endpoints of the segments and the value of the segment levels of the underlying piecewise constant function. The Bayesian Regression Curve (BRC) estimates the same data with a smoothing curve. However, in the original formulation, some estimators failed to properly determine the corresponding parameters. For example, the boundary estimator did not take into account the dependency among the boundaries and succeeded in estimating more than one breakpoint at the same position, losing segments.

**Results:**

We derived an improved version of the BPCR (called mBPCR) and BRC, changing the segment number estimator and the boundary estimator to enhance the fitting procedure. We also proposed an alternative estimator of the variance of the segment levels, which is useful in case of data with high noise. Using artificial data, we compared the original and the modified version of BPCR and BRC with other regression methods, showing that our improved version of BPCR generally outperformed all the others. Similar results were also observed on real data.

**Conclusion:**

We propose an improved method for DNA copy number estimation, mBPCR, which performed very well compared to previously published algorithms. In particular, mBPCR was more powerful in the detection of the true position of the breakpoints and of small aberrations in very noisy data. Hence, from a biological point of view, our method can be very useful, for example, to find targets of genomic aberrations in clinical cancer samples.

## Background

Lesions at DNA level represent the cause of cancer and of many congenital or hereditary disorders. The change of the number of copies of DNA in a genomic region is one of the most common aberrations. In normal cells each genomic segment is present in two copies, but, for example, in tumor cells the genome can present regions of deletions (copy number one or zero), gains (copy number three or four) or amplifications (copy number greater than four). Thus, in general, the DNA copy number along the genome can be represented as a piecewise constant function.

With microarray technology it is possible to simultaneously measure the copy number along the genome at hundred thousands of positions (see for example [1]). However, raw copy number data are generally very noisy. Hence, it is important to define a method which allows one to estimate the number of regions with different copy number, the endpoints of these regions (called *breakpoints*) and their copy number. Several methods have been developed to solve this issue. Many methods consider the log_2 _ratio of the data (the ratio is computed with respect to a normal reference sample) and model it as a normal random variable, since they assume that the errors are normally distributed. We can roughly subdivide all of these methods into two classes: those ones that estimate the copy numbers as a piecewise constant function and the others that estimate the copy numbers as a continuous curve. The methods belonging to the latter group are called smoothing methods.

Among the methods belonging to the first class, we can find the following. The Circular Binary Segmentation (CBS) approach is a recursive method in which the breakpoints are determined on the basis of a test of hypothesis, with null hypothesis that in the interval considered there is no change in copy number [2]. Picard et al. [3] used a piecewise constant regression model, where the parameters are estimated maximizing a penalized likelihood (i.e. the likelihood with the addition of a penalty function). This method is usually denoted with the abbreviation CGHseg. The GLAD method is another piecewise constant regression method, but in this case the parameters are estimated maximizing a weighted likelihood [4]. Fridlyand et al. [5] applied Hidden Markov Models (HMM), while Marioni et al. [6] defined an HMM method which takes into account the distance among the data points (BioHMM). Recently, Nilsson et al [7] derived a segmentation method based on total variation minimization, called Rendersome. It is optimized for gene expression data, but the authors affirm that it can be used also on copy number data.

Among the smoothing methods, Hsu et al. [8] used a wavelet regression method with Haar wavelet. Eilers and de Menez [9] applied a quantile smoothing regression (quantreg), with the solution found by minimizing a loss function based on the *L*_1 _norm, to obtain a flatter curve. Huang et al. [10] proposed smoothseg, i.e. a smooth segmentation method based on a doubly heavy-tailed random-effect model.

We propose a piecewise constant regression method, using Bayesian statistics, which appears appropriate when regions contain only few data points. The original version of the method (called Bayesian Piecewise Constant Regression, BPCR) was presented by Hutter [11,12]. In this paper we propose improved Bayesian estimators of the parameters involved and we apply the model to DNA copy number estimation. Finally, we compare our algorithm with some among the most cited or more recent methods, on artificial and real data.

Our method was implemented in R and is freely available at  or in Additional file 1. Furthermore, an R package will be soon available.

## Methods

In the first two subsections, we briefly describe the original Bayesian Piecewise Constant Regression method, explaining the hypothesis of the model and the estimation of its parameters with Bayesian regression. We emphasize the definitions of the original parameter estimators in order to show how we changed some of these estimators in the other subsections.

A brief explanation of the dynamic program for the computation of the estimators can be found in the Additional file 2 (more details can be found in [11,12]).

Regarding notations, we do not indicate explicitly the random variable to which a distribution is referred, if it is clear from the context. For example, *p*_*K*_(*k*) ≡ *p*(*k*) or *f*_***Y***, **M**_(***y***, ***μ***) ≡ *f*(***y***, ***μ***).

### Hypotheses of the model

Let ***Y ***∊ ℝ^*n *^be a random vector, such that each component (called *data point *or, if ***Y ***represents a quantity measured on part of the genome, *probe*) is conditionally normally distributed:

$$Y_{i}{|}_{{\tilde{\mu }}_{i}^{0},\sigma^{2}}\sim N({\tilde{\mu }}_{i}^{0},\sigma^{2}).$$

Suppose also that ***Y ***represents a noisy observation of a piecewise constant function, which consists of *k*_0 _horizontal segments. Then, the segment level at a generic position *i *(${\tilde{\mu }}_{i}^{0}$) does not assume different values for each *i*, but the data are divided into *k*_0 _intervals (with boundaries $0=t_{0}^{0}<t_{1}^{0}<\cdots <t_{k_{0}-1}^{0}<t_{k_{0}}^{0}=n$) where ${\tilde{\mu }}_{t_{q-1}+1}^{0}=...={\tilde{\mu }}_{t_{q}}^{0}=:\mu_{q}^{0}$ for each q = 1,...,*k*_0_. Hence, $\mu_{q}^{0}$ represents the level of the *q*^*th *^segment. Our goal is to estimate the levels $\mu^{0}=(\mu_{1}^{0},...,\mu_{k_{0}}^{0})$ of all the segments. In order to do that, we first need to estimate the number of the segments *k*_0 _and the partition of the data ***t***^0^. From a Bayesian point of view, **μ**^0^, **t**^0 ^and *k*_0 _are treated as random variables, hence we denote them with the corresponding upper case letters (**M**, ***T ***and *K*). Moreover, because of their randomness, we need to define a prior distribution for each of them to complete the model.

For the number of segments and the boundaries, we assume noninformative prior distributions:

(1) $$\begin{array}{cc} p(k)=\frac{1}{k_{\max}} & k\in K \end{array}$$

(2) $$\begin{array}{cc} p(t|k)=\frac{1}{\left(\begin{array}{c} n-1\\ k-1 \end{array}\right)} & t\in T_{k,n}, \end{array}$$

where K = {1,...,*k*_max_} and $T_{k,n}$ is the subspace of ${\mathbb{N}}_{0}^{k+1}$ such that *t*_0 _= 0, *t*_*k *_= *n *and *t*_*q *_∊ {1,...,*n *- 1} for all *q *= 1,...,*k *- 1, in an ordered way and without repetitions.

About **M**, we assume that all its components are mutually independent and identically normally distributed,

$$M{|}_{\nu ,\rho^{2},K=k}\sim N(\nu ,\rho^{2}I),$$

where ***ν ***∊ ℝ^*k*^, such that *ν*_*q *_= *ν *for each *q *= 1,...,*k*, and I ∊ ℝ^*k *× *k*^, such that I_*p*, *q *_= *δ*_*p*,*q *_for each *p*, *q *= 1,...,*k*.

Instead of these assumptions, we could assume a Cauchy distribution for each *Y*_*i *_or M_*q *_in order to model an observation whose noise has heavier tails, as previously done by Hutter [11,12].

### Original estimation: the BPCR method

The statistical procedure consists in a sequence of estimations due to the relationship among the parameters.

BPCR estimates the number of segments with the MAP (Maximum A Posteriori) estimate given the sample point ***y***,

(3) $$\hat{k}:=\underset{k\in K}{\arg\max}p(k|y),$$

and, given $\hat{k}$, also each boundary is estimated separately with its corresponding MAP estimate,

(4) $${\hat{t}}_{p}:=\underset{h\in \{p,...,n-(\hat{k}-p)\}}{\arg\max}\text{P}(T_{p}=h|y,\hat{k})$$

for all *p *= 1,...,$\hat{k}$ - 1. Finally, the *r*^*th *^moment of the level of the *m*^*th *^segment is estimated with its posterior mean. Since its computation needs the knowledge of the number of segments and the partition of the data, we replace them with the estimated ones,

(5) $${\hat{\mu }}_{m}^{r}:=E[{\text{M}}_{m}^{r}|y,\hat{t},\hat{k}]$$

for all *m *= 1,...,$\hat{k}$. When *r *= 1 and we assume that ***Y ***and **M **are normally distributed, the estimate turns out to be

(6) $${\hat{\mu }}_{m}=\frac{\rho^{2}\sum_{i=t_{m-1}+1}^{t_{m}}y_{i}+\sigma^{2}\nu }{(t_{m}-t_{m-1})\rho^{2}+\sigma^{2}},$$

for all *m *= 1,...,$\hat{k}$. When the sample contains only one segment, the Bayesian estimation of the posterior distribution of the levels should theoretically lead to a normal distribution, similar to a Dirac delta function centered at $\hat{\nu }$, since the levels can assume only one value from the data. In fact, in this case, if we estimate *ρ*^2 ^only using the data (without using any prior or other information), then this value will be close to zero (the variance of a constant random variable) and so the level will be estimated with $\hat{\nu }$, the mean of the data (see Equation (9)).

We can estimate the segment level ${\tilde{\text{M}}}_{s}$ at a generic position *s*, using the fact that it belongs to some segment *m *and in this segment it is equal to the corresponding M_*m*_. Then, summing over all the possible segments, we can compute its posterior distribution in the following way:

(7) $$f({\tilde{\mu }}_{s}|y,K=k_{0})=\sum_{m=1}^{k_{0}}\sum_{i=0}^{s-1}\sum_{j=s}^{n}f(\mu_{m},T_{m-1}=i,T_{m}=j|y,K=k_{0})$$

and the corresponding estimate of ${\tilde{\text{M}}}_{s}$ given $\hat{k}$ is

(8) $${\hat{\tilde{\mu }}}_{s}:=E[{\tilde{\text{M}}}_{s}|y,\hat{k}],$$

for all *s *= 1,...,*n*. The vector $\hat{\tilde{\mu }}$ is called *Bayesian Regression Curve *(*BRC*).

The probability distributions defined previously depend on the hyper-parameters *ν*, *ρ*^2 ^and *σ*^2 ^(respectively, the mean and the variance of the segment levels and the variance of the noise). Hutter [11,12] suggested the following estimators:

(9) $$\hat{\nu }:=\frac{1}{n}\sum_{i=1}^{n}Y_{i}=\bar{Y}$$

(10) $${\hat{\rho }}^{2}:=\frac{1}{n}\sum_{i=1}^{n}{\left(Y_{i}-\bar{Y}\right)}^{2}$$

(11) $${\hat{\sigma }}^{2}:=\frac{1}{2(n-1)}\sum_{i=1}^{n-1}{\left(Y_{i+1}-{\bar{Y}}_{i}\right)}^{2}.$$

### Improved estimators of the number of segments

To understand the real meaning of the MAP estimator $\hat{K}$, we need to introduce the theory of the construction of a generic Bayesian estimator.

In general, a Bayesian estimator is defined in the following way. Let us suppose that *Z *is a random variable whose distribution depends on an unknown parameter *θ*, which we want to estimate. Since we cannot exactly know the true value of the parameter, we consider it as a random variable Θ with a given prior probability distribution. In order to measure the goodness of the estimation, we define an error (or *loss function*) and we choose the estimator that minimizes the expected error given the sample ***Z***,

(12) $$\hat{\Theta }:=\arg\underset{\theta^{\prime }}{\min}E[err(\Theta ,\theta^{\prime })|Z].$$

The 0–1 error (defined as 1 -*δ*_*θ*, *θ'*_) is commonly used for a parameter which can assume only a discrete number of values. The estimator corresponding to this error is the MAP estimator,

(13) $$\begin{array}{lll} \arg\underset{\theta^{\prime }}{\min}E[1-\delta_{\Theta ,\theta^{\prime }}|Z] & = & \arg\underset{\theta^{\prime }}{\max}\sum_{\theta }\delta_{\theta ,\theta^{\prime }}p(\theta |Z)\\ & = & \arg\underset{\theta^{\prime }}{\max}p(\theta^{\prime }|Z). \end{array}$$

Obviously, if we use different types of errors, we can obtain different estimators. In the following, we will use $\hat{K}$ to denote any estimator of *K*, while $\hat{K}$_01 _to denote the parameter estimator $\hat{K}$ based on the 0–1 error.

Using the 0–1 error, we give the same penalty to each value different from the true value, whether it is close to or far away from the true one. To take into account the distance of the estimated value from the true one, we need to use other types of errors, which are based on different definitions of distance, such as,

(14) absolute error := |*θ *- *θ'*|

(15) squared error := (*θ *- *θ'*)^2^.

If the parameter *θ *∊ ℝ, then the estimators corresponding to these errors are the median and the mean of its posterior distribution, respectively. In our case, we denote these estimators of *k*_0 _with ${\hat{K}}_{1}$ and ${\hat{K}}_{2}$.

### Improved estimators of the boundaries

Similarly to the previous subsection, we derive alternative boundary estimators, by considering different types of errors. We denote the MAP boundary estimator defined in Equation (4) with ${\hat{T}}_{01}$.

#### Meaning of the estimator ${\hat{T}}_{01}$

The estimator ${\hat{T}}_{01}$ is defined in such a way that each component minimizes the 0–1 error of the corresponding boundary, separately. Explicitly, given the sample point ***y ***and the segment number *k*_0_, its estimate is

$${\hat{T}}_{01}=\left(0,\arg\underset{t_{1}\in T}{\max}p(t_{1}|y,K=k_{0}),...,\arg\underset{t_{k_{0}-1}\in T}{\max}p(t_{k_{0}-1}|y,K=k_{0}),n\right),$$

where T = {1,...,*n *- 1}. ${\hat{T}}_{01}$ may be regarded as an approximation of the Bayesian estimator that minimizes the error which counts the number of wrongly estimated boundaries:

(16) $$\text{sum }0\text{-}1\text{ error}=\sum_{p=1}^{k_{0}-1}\left(1-\delta_{t_{p}^{0},t_{p}}\right)=k_{0}-1-\sum_{p=1}^{k_{0}-1}\delta_{t_{p}^{0},t_{p}},$$

that is

(17) $${\hat{T}}_{\text{sum}}=\underset{t\in T_{k_{0},n}}{\arg\max}\sum_{p=1}^{k_{0}-1}p(t_{p}|K=k_{0},Y).$$

#### Definition of the estimator ${\hat{Τ}}_{\text{joint}}$

A problem of the latter estimator (Equation (17)) is its computational complexity, because it needs the computation of all the ordered combinations of the boundaries. On the other hand, there are two reasons for which ${\hat{T}}_{01}$ is not a suitable estimator of the boundaries. First, it does not take into account that the boundaries are dependent, because they have to be ordered, and second, in principle, it can have more than one component with the same value. As a consequence, a theoretically more correct way to estimate the boundaries is minimizing the 0–1 error with respect to the joint boundary probability distribution (this error is called *joint 0–1 error*). Then, given *k*_0 _and ***Y***, the boundary estimator becomes

(18) $${\hat{T}}_{\text{joint}}=\underset{t\in T_{k_{0},n}}{\arg\max}p(t|K=k_{0},Y).$$

#### Definition of the estimators $\hat{Τ}$_BinErr _and $\hat{Τ}$_BinErrAk_

We must notice that the estimators considered until now have the same length of the true vector of the boundaries. In practice, the number of segments *k*_0 _is unknown, so that we should use $\hat{k}$. As a consequence, if $\hat{k}$ is different from *k*_0_, then, strictly speaking, we cannot minimize the previous types of error because the vectors have different length.

A way to solve this issue is to map each boundary vector into a vector $\tau \in {\mathbb{R}}_{0}^{n+1}$ in the following way:

(19) $$t\mapsto \tau \text{ such that }\tau_{i}=\{\begin{array}{ll} 1 & \text{if }\exists p\text{ such that }t_{p}=i\\ 0 & \text{otherwise}. \end{array}$$

We denote with $TT_{k,n}$ the set of all the possible **τ **with *τ*_0 _= 1, *τ*_*n *_= 1 and *k *- 1 of the other components equal to 1.

Now, for the two new vectors ***τ***^0 ^and ***τ***, we define the following binary error,

(20) $$\text{binary error}=k_{0}-1-\langle \tau^{0},\tau \rangle =k_{0}-1-\sum_{i=1}^{n-1}\tau_{i}^{0}\tau_{i}.$$

Since the two-norm of the vectors involved is fixed, minimizing (20) is the same as minimizing the Euclidean distance between the two vectors or the sum 0–1 error. Furthermore, error (20) is consistent with the Russell-Rao dissimilarity measure defined on the space of the binary vectors. Its corresponding estimator is

(21) $${\hat{Τ}}_{\text{BinErr}}:=\underset{\tau^{\prime }\in TT_{k_{0},n}}{\arg\min}\text{E}\left[k_{0}-1-\sum_{i=1}^{n-1}\tau_{i}{\tau^{\prime }}_{i}|Y,k_{0}\right]=\underset{\tau^{\prime }\in TT_{k_{0},n}}{\arg\max}\text{E}\left[\sum_{i=1}^{n-1}\tau_{i}{\tau^{\prime }}_{i}|Y,k_{0}\right].$$

Since we do not know the real value of *k*_0_, we should replace it with $\hat{k}$ to compute Equation (21). Doing this, we could amplify the error of the boundary estimation because of the addition of the error of the segment number estimation. A way to attenuate this issue is to integrate out the number of segments in the conditional expected value. Then the estimator becomes

(22) $${\hat{Τ}}_{\text{BinErrAk}}:=\underset{\tau^{\prime }\in TT_{\hat{k},n}}{\arg\max}\text{E}\left[\sum_{i=1}^{n-1}\tau_{i}{\tau^{\prime }}_{i}|Y\right].$$

#### Improved regression curve

As we saw in the previous subsections, there are cases in which the estimation of a parameter of our interest can be made independently of other parameters by integration. The computation of the BRC (see Equations (7) and (8)) suggests to average also over the number of segments by considering the posterior probability of ${\tilde{\text{M}}}_{s}$, given only the sample point ***y***,

(23) $${\hat{\tilde{\mu }}}_{s}:=E[{\tilde{\text{M}}}_{s}|y].$$

Unfortunately, the computation of this quantity requires time $O(n^{2}k_{\max}^{2})$ (see section "The dynamic program" in Additional file 2), hence it could be a problem with samples of big size. This new type of ${\tilde{\text{M}}}_{s}$ estimation is referred to as *Bayesian Regression Curve Averaging over k *(*BRCAk*).

The same procedure cannot be applied for the level estimation, because in that case we need to know the partition of the whole interval.

### Properties of the hyper-parameter estimators and definition of new estimators

In order to study the properties of the hyper-parameter estimators defined in Equations (9), (11) and (10), first we need to compute the moments of the data $Y{|}_{\nu ,\rho^{2},\sigma^{2}}$. In the following, we will denote with *n*_*q *_the number of data points in the *q*^*th *^segment.

At first, let us consider only the data which belong to the *q*^*th *^segment. From the hypothesis of the model, we know that

$$\begin{array}{lll} Y_{j}{|}_{M_{q},\sigma^{2}} & \sim & \begin{array}{cc} N({\text{M}}_{q},\sigma^{2}) & j=t_{q-1}+1,...,t_{q} \end{array}\\ M_{q}{|}_{\nu ,\rho^{2}} & \sim & N(\nu ,\rho^{2}), \end{array}$$

hence the marginal distribution of any two data points *Y*_*i *_and *Y*_*j *_belonging to the *q*^*th *^segment is N(**ν**, Σ), where

(24) $$\Sigma =\left[\begin{array}{cc} \sigma^{2}+\rho^{2} & \rho^{2}\\ \rho^{2} & \sigma^{2}+\rho^{2} \end{array}\right].$$

It follows that the covariance between two data points, which belong to the same segment, is

(25) Cov(*Y*_*i*_, *Y*_*j*_|*ν*, *ρ*^2^, *σ*^2^) = *ρ*^2 ^*i *≠ *j*,

and

(26) *E*[*Y*_*j*_|*ν*, *ρ*^2^, *σ*^2^] = *ν*

(27) Var(*Y*_*j*_|*ν*, *ρ*^2^, *σ*^2^) = *σ*^2 ^+ *ρ*^2^,

for each *j *= 1,...,*n*, independently of the segment to which it belongs.

Furthermore, from the hypotheses of the model, given the segmentation ***t***^0^, data points belonging to different segments are independent.

#### Expected value and variance of the estimator $\hat{\nu }$

The estimator of *ν *is defined as $\hat{\nu }=\bar{Y}$ (see Equation (9)). From Equation (26), we can see that this estimator is unbiased and its variance turns out to be

(28) $$\text{Var}[\bar{Y}]=\frac{\sigma^{2}}{n}+\rho^{2}\frac{\sum_{p=1}^{k_{0}}n_{p}^{2}}{n^{2}}.$$

Hence, the variance is always greater than $O\left(\frac{\rho^{2}}{k_{0}}\right)$, even if we use a denser sampling, i.e. we augment the number of data points in the interval in which we are estimating the piecewise constant function.

#### New definition of the estimator $\hat{\sigma^{2}}$ and its expected value

A circular version of the *σ*^2 ^estimator defined in Equation (11) is

(29) $$\hat{\sigma^{2}}:=\frac{1}{2n}\sum_{i=1}^{n}{\left(Y_{i+1}-Y_{i}\right)}^{2},$$

where *Y*_*n*+1 _:= *Y*_1_. Using the values of the moments of the data points, its expected value is

(30) $$E\hat{\left[\sigma^{2}\right]}=\sigma^{2}+\rho^{2}\frac{k_{0}}{n}I_{\left\{k_{0}\geq 2\right\}},$$

where we considered two cases in the computation: (a) when *k*_0 _= 1, *Y*_1 _and *Y*_*n *_belong to the same segment (thus they are dependent), (b) when *k*_0 _≥ 2, we supposed that the first and the last segments have different levels and so *Y*_1 _and *Y*_*n *_are independent. If the first and the last segments had the same level, then the two segments would be joined together and thus *Y*_1 _and *Y*_*n *_would be dependent. In this case, the expected value would be the same but with *k*_0 _- 1 instead of *k*_0_, since the number of segments would be *k*_0 _- 1. In any case, for *k*_0 _= 1, the estimator $\hat{\sigma^{2}}$ is unbiased, while for *k*_0 _≪ *n *but *k*_0 _≠ 1, $\hat{\sigma^{2}}$ is almost unbiased.

#### Expected value of the estimator $\hat{\rho^{2}}$

The expected value of the estimator $\hat{\rho^{2}}$ (defined in Equation (10)) is

(31) $$E[\hat{\rho^{2}}]=\sigma^{2}\left(1-\frac{1}{n}\right)+\rho^{2}\left(1-\frac{\sum_{p=1}^{k_{0}}n_{p}^{2}}{n^{2}}\right).$$

Note that when *k*_0 _= 1 (i.e. having only one segment), $E[\hat{\rho^{2}}]=\sigma^{2}\left(1-\frac{1}{n}\right)$. In this degenerate case, the variance of the segment levels *ρ*^2 ^should be estimated with zero but $\hat{\rho^{2}}$ estimates it with the variance of the data points.

Moreover, since $\sum_{p=1}^{k_{0}}n_{p}^{2}\geq n$ (the equality holds only when *k*_0 _= *n*), we obtain that

(32) $$\sigma^{2}\left(1-\frac{1}{n}\right)\leq E[\hat{\rho^{2}}]\leq \left(1-\frac{1}{n}\right)\left(\sigma^{2}+\rho^{2}\right).$$

Hence, if *n *is large the expected value is between *σ*^2 ^and *σ*^2 ^+ *ρ*^2^, so that, if *ρ*^2 ^≪ *σ*^2^, the estimator is almost unbiased for *σ*^2 ^(instead of *ρ*^2^).

#### Definition of alternative estimator of *ρ*^2^: $\hat{\rho_{1}^{2}}$

Since the covariance between data points belonging to the same segment is *ρ*^2^, we could try to use a circular version of the estimator of the autocovariance of a stationary time series

(33) $$\hat{\rho_{1}^{2}}:=\frac{1}{n}\sum_{i=1}^{n}(Y_{i}-\bar{Y})(Y_{i+1}-\bar{Y}),$$

where *Y*_*n*+1 _:= *Y*_1_. The expected value of the estimator turns out to be

(34) $$E[\hat{\rho_{1}^{2}}]=\{\begin{array}{ll} -\frac{\sigma^{2}}{n} & \text{if }k_{0}=1\\ \frac{\rho^{2}}{n}\left(n-k_{0}-\frac{\sum_{p=1}^{k_{0}}n_{p}^{2}}{n}\right)-\frac{\sigma^{2}}{n} & \text{if }k_{0}\geq 2. \end{array}$$

In the computation we considered two cases: *k*_0 _= 1 and *k*_0 _≥ 2. When *k*_0 _= 1, *Y*_1 _and *Y*_*n *_belong to the same segment and so they are dependent; when *k*_0 _≥ 2, we suppose that the first and the last segment have not the same level value and so *Y*_1 _and *Y*_*n *_are independent. If *k*_0 _≥ 2 and the first and the last segment had the same level value (event with a very low probability), then the first and the last segments would be joined together and so *Y*_1 _and *Y*_*n *_would be dependent. In this case, the expected value of the estimator would have the same formula, but with *k*_0 _- 1 instead of *k*_0_.

We can observe that, when *k*_0 _= 1, the expected value is negative, while, when *k*_0 _≥ 2, it can be negative or positive. Moreover, the coefficient of *σ*^2 ^is $-\frac{1}{n}$ and so this addendum does not contribute so much to the unbiasedness of the estimator.

The negativity of the expected value happens because the estimator is a generic estimator of the covariance and, in general, this quantity can be negative. To prevent the negativity of the estimator, we can use its absolute value. In this way, when the quantity in (33) is negative, we use the same estimator but with the sign changed in one of the factors of each product, $\hat{\rho_{1}^{2}}=\frac{1}{n}\sum_{i=1}^{n}\left(Y_{i}-\bar{Y})(\bar{Y}-Y_{i+1}\right)$. Hence, the meaning of the estimator is the same. We are interested only in the absolute value of the estimate and not in its sign. In fact, we already know that the correlation is positive and the negativity of the estimate is due only to the property of the estimator. Our final definition of the estimator is then

(35) $$\hat{\rho_{1}^{2}}:=\frac{1}{n}\left|\sum_{i=1}^{n}\left(Y_{i}-\bar{Y})(Y_{i+1}-\bar{Y}\right)\right|.$$

## Results and discussion

In this section we show and discuss results obtained on both the simulated and the real data. We used the simulated data with a twofold aim. The first was to choose empirically the best estimators among those proposed in the previous section. The second was to compare the original version of BPCR/BRC and their modified versions with each other and with other existing methods estimating DNA copy number value [2-10]. On the basis of the results, we selected the best modified version of BPCR, called mBPCR, and of BRC.

Finally, we compared the performance of mBPCR, CBS, CGHseg, GLAD, HMM, BioHMM and Rendersome on the real data.

### Simulation description

In the comparisons, we used several types of artificial data. We call *sample *a sequence of data which represents the copy number data of a genomic region, we call *dataset *a set of samples, while *collection *a set of datasets.

**Figure 1:**
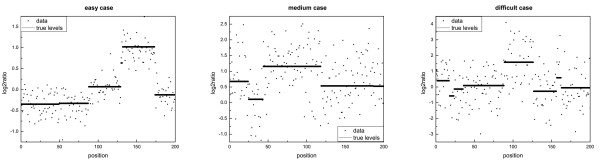
**Example of simulated data**. The simulated data in the figure represent an easy, medium and difficult case, respectively.

In order to experimentally evaluate the behavior of all the estimators proposed, we used the artificial datasets sampled from the priors, defined in the hypotheses of the model. We always chose *ν *= 0.2, while we changed the values of *σ*^2 ^and *ρ*^2 ^for each dataset, in order to study different situations of noise (some examples of data are in Figure 1 and the corresponding estimated profiles obtained applying several methods are in Figure S.1 in Additional file 2). The most problematic cases were the ones with *ρ*^2 ^<*σ*^2 ^(i.e. when the variance of the noise was higher than the variance of the segment levels), because in these cases it was hard to identify the true profile of the levels. We always used *n *= 200, similar to the mean number of probes of a small chromosome in the Affymetrix GeneChip Mapping 10K Array (hence it represented a difficult case due to the small sample size), and *k*_max _= 40, in order to have at least 5 probes per segment on average.

Sometimes we needed datasets where all samples had the same true profile of the segment levels (i.e. *K*, ***T ***and ***M ***were sampled one time and only the noise varied in all samples). This type of dataset is called *dataset with replicates*. Otherwise, the dataset is called *without replicates *(i.e. each time we sampled *K*, ***T***, ***M ***and added the noise to the profile). The number of samples per dataset was 100, for datasets with replicates, and 300 otherwise. We considered datasets with replicates in order to be able to compare the goodness of different types of estimations for a given profile.

We also compared the behavior of our boundary estimators using the artificial dataset already employed in [13], where three methods for copy number estimation were examined. This dataset contained 500 samples consisting of 20 chromosomes, each of 100 probes, which emulated the real copy number data. This dataset is referred to as *Simulated Chromosomes*.

To assess the performance of the several methods, we used three types of artificial datasets. The first type consisted of four datasets with replicates used in the comparison among the estimators. This collection of datasets is called *Cases*.

The second type consisted of datasets adapted from the datasets used in [14] to compare several methods for copy number estimation. In these datasets, each sample was an artificial chromosome of 100 probes, where the copy number value was zero apart from the central part where there was an aberration. The Authors considered several widths of aberration: 40, 20, 10 and 5 probes. The noise was always distributed as N(0, 0.25^2^), while the signal to noise ratio (SNR) was 4, 3, 2 or 1. The SNR was defined as the ratio between the height of the aberration and the standard deviation of the noise. The data of the paper consisted of datasets of 100 samples for each combination of width and SNR.

We defined our datasets in the following way. For a fixed SNR value, we constructed a chromosome with four aberrations of width of 40, 20, 10 and 5 probes, respectively, by joining the corresponding four types of chromosome of the previous datasets. This collection of datasets is called *Four aberrations*. In the following, we will consider only the datasets with SNR = 3 (medium noise) and SNR = 1 (high noise).

The third type of dataset used was the *Simulated Chromosomes *dataset.

### Comparison among the estimators on simulated data

In this subsection, we present how we selected the best estimators among those proposed in the Section Methods, on the basis of their results obtained on the artificial datasets. The comparisons were accomplished using both the true and the estimated values of the other parameters involved in the estimation.

#### Comparison among the hyper-parameter estimators

We applied the hyper-parameter estimators on 8 datasets without replicates, considering different values for *σ*^2 ^and *ρ*^2^. To evaluate the behavior of the hyper-parameter estimators in all these cases, for each dataset we computed the (estimated) Mean Square Error, MSE, with respect to the true value of the parameter (Table 1). To measure the accuracy of the estimators, we used the estimated mean relative error over all datasets (Table S.1 in Additional file 2).

**Table 1:** Comparison among the hyper-parameter estimators

	*σ*^2 ^= 0.1	*σ*^2 ^= 0.3	*σ*^2 ^= 0.5	*σ*^2 ^= 0.5	*σ*^2 ^= 0.5	*σ*^2 ^= 0.7	*σ*^2 ^= 1	*σ*^2 ^= 1.2
	*ρ*^2 ^= 0.5	*ρ*^2 ^= 0.05	*ρ*^2 ^= 0.02	*ρ*^2 ^= 0.05	*ρ*^2 ^= 0.1	*ρ*^2 ^= 0.5	*ρ*^2 ^= 0.05	*ρ*^2 ^= 0.5
${\text{MSE}}_{\hat{\nu }}$	0.0904	0.0091	0.0059	0.0094	0.021	0.067	0.0169	0.0729
${\text{MSE}}_{{\hat{\sigma }}^{2}}$	0.0042	0.0014	0.0041	0.0036	0.0037	0.0114	0.0123	0.0272
${\text{MSE}}_{{\hat{\rho }}^{2}}$	0.0633	0.0871	0.2508	0.2404	0.2426	0.4271	0.9921	1.3254
${\text{MSE}}_{{\hat{\rho }}_{1}^{2}}$	0.068	0.0009	0.0008	0.0014	0.0047	0.0593	0.0024	0.0623

The table shows the estimated mean square error of the estimators $\hat{\nu }$, ${\hat{\sigma }}^{2}$, ${\hat{\rho }}^{2}$ and ${\hat{\rho }}_{1}^{2}$ applied to datasets without replicates, for different values of *σ*^2 ^and *ρ*^2^. The table shows that ${\text{MSE}}_{\hat{\nu }}$ increased with *ρ*^2 ^and ${\text{MSE}}_{{\hat{\sigma }}^{2}}$ increased with *σ*^2^. The estimator ${\hat{\rho }}_{1}^{2}$ was generally more accurate than ${\hat{\rho }}^{2}$ with respect to the MSE error measure.

From the results, we can deduce that ${\hat{\sigma }}^{2}$ is a good estimator because it was quite precise in all situations, while $\hat{\nu }$ was sometimes poor but in general acceptable. About the *ρ*^2 ^estimation, it is better to use ${\hat{\rho }}_{1}^{2}$ than ${\hat{\rho }}^{2}$, when the variance of the noise is higher than the variance of the levels. Otherwise, it seems better to use ${\hat{\rho }}^{2}$ because it does not underestimate *ρ*^2 ^(see Section Methods).

#### Comparison among the segment number estimators

We evaluated the quality of the estimators of the number of segments, using datasets with and without replicates for different values of the hyper-parameters *σ*^2 ^and *ρ*^2^. The estimations were made using either the true values of the hyper-parameters or the estimated ones. In this way, we could also observe the behavior of the boundary estimators without the influence of the hyper-parameter estimation.

Comparing the absolute, squared and 0–1 errors, we found that ${\hat{K}}_{2}$ generally had the lowest upper bound (or its upper bound is very close to the lowest one) of the confidence interval at level 95%, for any type of error and any type of value of the parameter *ρ*^2 ^(see, for example, Figures S.2 and S.3 in Additional file 2). Moreover, all estimators always had a similar confidence interval of the 0–1 error, while using ${\hat{\rho }}^{2}$, in most cases the upper bound of the confidence interval of the absolute and the squared error was lower than using ${\hat{\rho }}_{1}^{2}$. All these results support the suggestion to use ${\hat{K}}_{2}$ with ${\hat{\rho }}^{2}$.

We should also observe that in general $\hat{K}$_01 _underestimates *k*_0_, while ${\hat{K}}_{1}$ and ${\hat{K}}_{2}$ overestimate it. In addition, the percentage of the underestimations increases using ${\hat{\rho }}^{2}$.

#### Comparison among the boundary estimators

We compared the boundary estimators on the same datasets, previously used for the estimators of the number of segments, with both the estimated and the true value of the parameters involved. The following errors were taken into account: the sum 0–1 error, the joint 0–1 error and the binary error, defined in Section Methods, and the average square error,

(36) $$\text{aver}\text{. square error}:=\frac{1}{k-1}\sum_{i=1}^{k-1}\underset{j=1,...,k-1}{\min}{\left(t_{j}-{\hat{T}}_{i}\right)}^{2},$$

which corresponds to the mean square error over the whole vector of estimated boundaries. As observed in Section Methods, when the estimated segment number is used in the estimation of the boundaries, we are only able to compute the binary error, because it does not require that the vector of estimated boundaries has the same length as the vector of the true boundaries.

We found (see, for example, Table 2 and Table S.2 in Additional file 2) that the best estimators were ${\hat{T}}_{BinErr}$, ${\hat{T}}_{BinErrAk}$ and ${\hat{T}}_{joint}$, but it seemed that ${\hat{T}}_{BinErr}$, ${\hat{T}}_{BinErrAk}$ were slightly better than ${\hat{T}}_{joint}$, when we estimated the parameters. Moreover, between these ones, we preferred the latter, because its computation depended less on the estimation of *k*_0 _and thus it was more stable. In addition, in case of *σ*^2 ^> *ρ*^2^, we observed that there was a great difference between the errors obtained using ${\hat{\rho }}^{2}$ and ${\hat{\rho }}_{1}^{2}$: using the latter, the errors were significantly lower (Table 2).

**Table 2:** Comparison among the several boundary estimators

	*σ*^2 ^= 0.1	*σ*^2 ^= 0.3	*σ*^2 ^= 0.5	*σ*^2 ^= 1.2
**method**	*ρ*^2 ^= 0.5	*ρ*^2 ^= 0.05	*ρ*^2 ^= 0.1	*ρ*^2 ^= 0.5
${\hat{T}}_{01}$, ${\hat{\rho }}^{2}$	11.5967 ± 0.4562	18.0933 ± 0.6388	18.2 ± 0.6177	17.6833 ± 0.5781
${\hat{T}}_{joint}$, ${\hat{\rho }}^{2}$	8.8667 ± 0.3329	17.6333± 0.6224	17.4833± 0.5898	16.6167 ± 0.5367
${\hat{T}}_{BinErr}$, ${\hat{\rho }}^{2}$	8.6833 ± 0.3437	17.3833 ± 0.6219	17.2467 ± 0.5983	15.8967 ± 0.5278
${\hat{T}}_{BinErrAk}$, ${\hat{\rho }}^{2}$	8.7267 ± 0.3449	17.3933 ± 0.6226	17.2567 ± 0.5978	15.9133 ± 0.5303

${\hat{T}}_{01}$, ${\hat{\rho }}_{1}^{2}$	11.4767 ± 0.4532	15.2867 ± 0.5325	15.6867 ± 0.536	16.1933 ± 0.5301
${\hat{T}}_{joint}$, ${\hat{\rho }}_{1}^{2}$	8.7933 ± 0.3294	13.73 ± 0.4731	13.81 ± 0.4691	14.1667 ± 0.451
${\hat{T}}_{BinErr}$, ${\hat{\rho }}_{1}^{2}$	8.47 ± 0.3372	13.0567 ± 0.4635	13.18 ± 0.4725	13.2367 ± 0.4344
${\hat{T}}_{BinErrAk}$, ${\hat{\rho }}_{1}^{2}$	8.4567 ± 0.3361	13.0467 ± 0.4676	13.08 ± 0.4695	13.2233 ± 0.4361

The table shows the estimated mean binary errors ± standard deviation of the estimators of ***t***^0 ^applied to datasets with replicates for different values of *σ*^2 ^and *ρ*^2^. We always used ${\hat{K}}_{2}$, $\hat{\nu }$, ${\hat{\sigma }}^{2}$ and both *ρ*^2 ^estimators as estimators of the other parameters involved. The estimators ${\hat{T}}_{BinErr}$ and ${\hat{T}}_{BinErrAk}$ always had the lowest errors. When *σ*^2 ^> *ρ*^2 ^the error was lower by using ${\hat{\rho }}_{1}^{2}$, otherwise the two *ρ*^2 ^estimators gave similar errors.

To choose between the estimators ${\hat{T}}_{BinErrAk}$ and ${\hat{T}}_{joint}$, we performed the segment level estimation, considering both *ρ*^2 ^estimators. Then, we computed the MSE of the estimated segment level per probe and its upper bound of the confidence interval at level 95% (the corresponding graphs regarding some datasets can be found in Figure S.5 in Additional file 2). The results showed that, for a given estimator of *ρ*^2^, in general ${\hat{T}}_{BinErrAk}$ was better than ${\hat{T}}_{joint}$ and the upper bound of the confidence interval of the MSE of the former estimator was lower than that one of the latter. Moreover, using ${\hat{\rho }}_{1}^{2}$ the error was generally lower. 

In addition, we compared the behavior of our boundary estimators on dataset *Simulated Chromosomes*. To assess the goodness of their estimation, we measured the sensitivity (proportion of true breakpoints detected) and the false discovery rate (FDR, i.e. proportion of false estimated breakpoints among the estimated ones), while to assess the influence of the boundary estimation on the profile estimation, we calculated the sum of squared distance (SSQ), the median absolute deviation (MAD) and the accuracy (proportion of probes correctly assigned to an altered or unaltered state). The sensitivity and the FDR were computed not only looking at the exact position of the breakpoints (w = 0), but also accounting for a neighborhood of 1 or 2 probes around the true positions (w = 1, 2). We also computed the accuracy inside and outside the aberrations separately, since the samples of dataset *Simulated Chromosomes *contained only few small copy number changes and thus the accuracy depended more on the correct estimation/classification of the probes in the "normal" regions. A more detailed explanation of these measures can be found in [13].

Since we estimated ${\hat{\sigma }}^{2}$ = 0.026 and ${\hat{\rho }}_{1}^{2}$ = 0.031 (using the whole dataset), we expected that using ${\hat{\rho }}^{2}$ we should obtain better results because *σ*^2 ^was lower than *ρ*^2 ^and indeed the results obtained with ${\hat{\rho }}^{2}$ were slightly better than the others (see Table 3 and Figure S.6 in Additional file 2). Moreover, we found in general that ${\hat{T}}_{BinErrAk}$ had the highest sensitivity but also a higher FDR than ${\hat{T}}_{01}$ (Figure S.6 in Additional file 2). This was due to the fact that, although the estimated number of segments was the same, ${\hat{T}}_{01}$ could estimate some breakpoints with the same position, reducing the total number of breakpoints and so reducing the FDR. We can see in Table 3 that the false estimated breakpoints did not negatively influence the profile estimation. In fact, the false breakpoints are often used by the algorithm in two ways: either to divide a long segment into two or more segments with close levels or, if it is difficult to determine the position of a breakpoint, to add, before or after the aberration, a segment of one point with the value of the level between zero and the aberration level. Overall, ${\hat{T}}_{BinErrAk}$ with ${\hat{\rho }}^{2}$ performed best on this dataset.

**Table 3:** Comparison among the boundary estimators on *Simulated Chromosomes*

**method**	**SSQ**	**MAD**	**accuracy**	**accuracy inside aberrations**	**accuracy outside aberrations**
${\hat{T}}_{01}$, ${\hat{\rho }}^{2}$	14.23	0.00877	0.889	0.961	0.883
${\hat{T}}_{joint}$, ${\hat{\rho }}^{2}$	2.22	0.00840	0.904	0.992	0.892
${\hat{T}}_{BinErrAk}$, ${\hat{\rho }}^{2}$	1.70	0.00733	0.936	0.992	0.932

${\hat{T}}_{01}$, ${\hat{\rho }}_{1}^{2}$	9.74	0.00952	0.881	0.960	0.877
${\hat{T}}_{joint}$, ${\hat{\rho }}_{1}^{2}$	2.67	0.00970	0.882	0.993	0.867
${\hat{T}}_{BinErrAk}$, ${\hat{\rho }}_{1}^{2}$	1.85	0.00781	0.929	0.993	0.920

This table shows the comparison among the error measures for profile estimation, obtained on dataset *Simulated Chromosomes*, for all estimators of ***t***^0 ^(apart ${\hat{T}}_{BinErr}$). We always used ${\hat{K}}_{2}$, $\hat{\nu }$, ${\hat{\sigma }}^{2}$ and both *ρ*^2 ^estimators as estimators of the other parameters involved. The estimator ${\hat{T}}_{BinErrAk}$ always had the lowest SSQ and MAD errors and the highest accuracy both inside and outside the regions of aberration. Using ${\hat{\rho }}^{2}$ the performance was slightly better, because *σ*^2 ^<*ρ*^2^.

In conclusion, we suggest to use ${\hat{T}}_{BinErrAk}$, even if ${\hat{T}}_{joint}$ is also a quite good estimator in some cases. Regarding the estimation of *ρ*^2^, it seems that it is better to use ${\hat{\rho }}_{1}^{2}$ in presence of high noise.

#### Comparison among the regression curves

We compared the estimation of the levels of BRC with the one of BRCAk, also taking into account the influence of the different estimators of the parameters on the final results. To valuate the performance of the methods, we used the root mean square error (RMSE) per probe and per sample, computed with respect to the true profile of the levels. For this purpose, we necessarily needed datasets with replicates. Using BRCAk we generally obtained a better or equal result with respect to the BRC (see, for example, Figures S.7 and S.8 in Additional file 2). Moreover, we observed that, using BRC, it was better to estimate the segment number with ${\hat{K}}_{1}$ or ${\hat{K}}_{2}$.

Note that we still have to solve the problem to determine which is the best estimator of *ρ*^2^. In most cases, the profile obtained by using ${\hat{\rho }}_{1}^{2}$ was better than using ${\hat{\rho }}^{2}$ (for example, see the plots at the bottom of Figure S.9 in Additional file 2). This is due to the fact that sometimes ${\hat{\rho }}_{1}^{2}$ slightly underestimated *ρ*^2^, leading to overfitting. Still we recommend to use ${\hat{\rho }}_{1}^{2}$, even if it could lead to a slight overfitting especially in the case of few segments.

### Comparison with other methods on simulated data

In this subsection we compare the original and modified versions of BPCR and BRC, with other existing methods for genomic copy number estimation [2-10].

#### Error measures used in the comparison

We used two different measures to examine the behavior of the different methods on the collections *Cases *and *Four aberrations*: the root mean square error (for both) and the ROC curve (only for the latter). Each point of the ROC curve has as coordinates the false positive rate (FPR) and the true positive rate (TPR) for a certain threshold. The TPR is defined as the fraction of probes in the true aberrations whose estimated value is above the threshold considered, while the FPR consists in the fraction of probes outside the true aberrations whose estimated value is above the threshold. Hence, the ROC curve measures the accuracy of the method in the detection of the true aberrations.

Instead, the evaluation of the several methods on dataset *Simulated Chromosomes *was accomplished using the error measures described in [13], already used in the study of the boundary estimators.

Before showing the results, we need to remember that some methods estimate the copy numbers as a piecewise constant function, while other algorithms estimate them as a continuous curve. Hence, BPCR was compared to the former group of methods, while the Bayesian regression curves to the latter. Since some error measures of [13] suppose that the estimated profile is piecewise constant, we applied only the former group of methods on dataset *Simulated Chromosomes*.

#### The piecewise constant estimation

**Figure 2:**
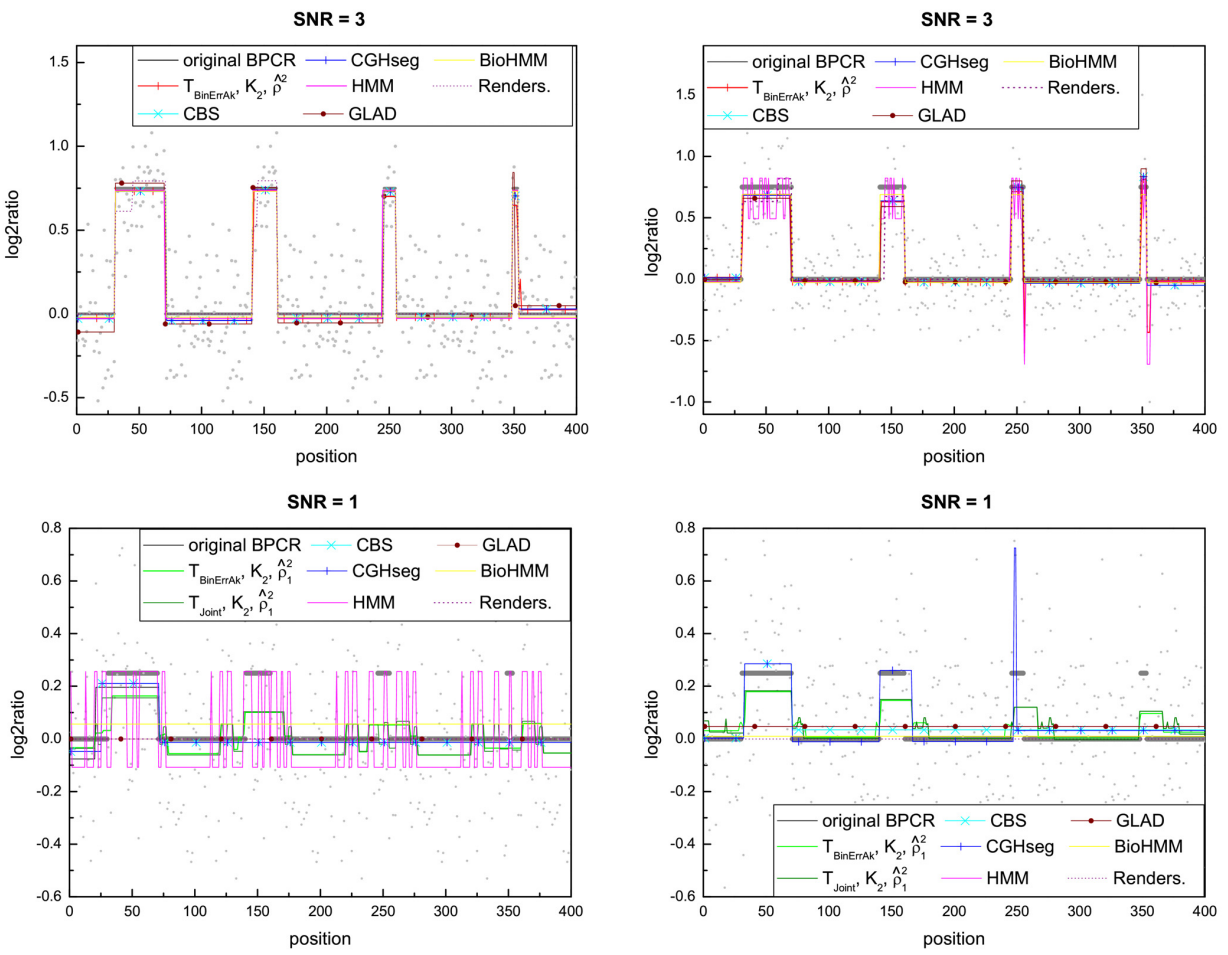
**Example of estimated piecewise constant profiles**. The plots show the differences in the level estimation among the piecewise constant methods on samples with SNR = 3 and SNR = 1: some are unable to identify the small aberrations in presence of high noise. In each graph, the grey segments represent the true profile.

We compared the original and the modified versions of BPCR with CBS [2], CGHseg [3], GLAD [4], HMM [5], BioHMM [6] and Rendersome [7]. For thoroughness, in the modified versions of BPCR, we used both ${\hat{\rho }}^{2}$ and ${\hat{\rho }}_{1}^{2}$ as estimators of *ρ*^2^, ${\hat{K}}_{2}$ as estimator of *k*_0 _and both ${\hat{T}}_{joint}$ and ${\hat{T}}_{BinErrAk}$ as estimators of the boundaries. We used ${\hat{\rho }}^{2}$ when the noise was low (*σ*^2 ^<*ρ*^2^) and otherwise ${\hat{\rho }}_{1}^{2}$. Examples of estimated profiles can be found in Figure 2.

In general, in presence of medium noise, the GLAD method performed worst, since it had a high error in the level estimation of the small peaks, while, for high noise, often both GLAD and Rendersome failed to detect the aberrations (Figures S.12 and S.13 in Additional file 2). The CGHseg method did not usually exhibit an appropriate level estimation except sometimes for segments of large width (for example in Figure S.11 in Additional file 2). This is due to the fact that CGHseg estimates the level of a segment with the arithmetic mean of the data points in the segment and this estimator performs poorly if the segment contains few data points and the breakpoint estimation is not accurate. The CBS method, in general, performed quite well, but it was unable to detect aberrations of small width, especially when the noise was high (Figure S.13 in Additional file 2).

On the collection *Cases *and the dataset of *Four aberrations *with SNR = 3, the RMSE plots and the ROC curves of the HMM method showed that it generally estimated the profile well, but sometimes it exhibited high errors near breakpoint positions (see, for example, Figure S.11 in Additional file 2), likely because it was unable to determine the true position of the breakpoints precisely. Moreover, on the dataset with SNR = 1, we recognized the true issues of the estimation with HMM. The RMSE plot showed that it had a high error outside the regions of the aberrations, while inside these regions the error was always more or less the same. Hence, it often failed also in the estimation of the largest aberration, the easiest one to detect (see the corresponding errors in Figure S.13 in Additional file 2). The reason of this behavior of the RMSE is the following. The method estimated the true profile either with only one segment, or more often with a profile consisting of a lot of small segments, but all with the same level. Since in the latter case the estimated levels were close to that one of the true aberrations, the RMSE was low in the regions of the aberrations but high outside them. However, the estimated profiles were not similar to the true one. In presence of medium noise (SNR = 3), the method BioHMM was more precise than HMM in the determination of the breakpoint positions and in the level estimation (Figure S.11 in Additional file 2), while for high noise it behaved similarly to HMM (Figure S.13 in Additional file 2).

In general we found that, when *σ*^2 ^<*ρ*^2 ^(when *σ*^2 ^> *ρ*^2^), the version of BPCR with ${\hat{T}}_{BinErrAk}$ and ${\hat{\rho }}^{2}$ (${\hat{\rho }}_{1}^{2}$) generally gave the best estimation compared to the other versions of BPCR and to the other methods (Figures S.10, S.11, S.12 and S.13 in Additional file 2). In the following, we will call this modified version of BPCR, mBPCR.

Only on the dataset with SNR = 1, we could not choose the "best" method, because this case showed the limits of all the methods considered. The problem regarding the modified versions of BPCR was essentially the estimation of the number of segments. The ROC curves (Figure S.12 in Additional file 2) of the modified versions of BPCR with ${\hat{\rho }}_{1}^{2}$ were the closest to the left and the top sides of the box, while the RMSE plot (Figure S.13 in Additional file 2) showed that these methods were the best methods in the estimation of the levels inside the aberrations, but not outside them. In general, in case of very high noise, all the modified versions of BPCR well detected the aberrations, but had problems in the estimation of the profile outside them because of the poor estimation of the number of segments. In fact, ${\hat{K}}_{2}$ tends to overestimate the number of segments and this problem worsens using ${\hat{\rho }}_{1}^{2}$. In conclusion, in a situation with very high noise, using ${\hat{\rho }}_{1}^{2}$ the BPCR methods detect better the small segments, but, at the same time, the large ones are divided in small segments. On the other hand, using ${\hat{\rho }}^{2}$, smaller segments are not detected and are joined to the closest large segment.

Finally, the comparison performed on dataset *Simulated Chromosomes *showed that CBS and mBPCR better estimated the profiles (see Table 4). Regarding the breakpoint error measures (see Figure S.14 in Additional file 2), we found that mBPCR had the highest sensitivity (hence, it was the best method in determining the exact position of the breakpoints), but also a higher FDR than CBS. We have already explained in the previous subsection the possible reason of the high FDR of mBPCR and we can observe again that this fact did not influence negatively the profile estimation (see the SSQ error in Table 4). The GLAD method showed a low sensitivity and low FDR, apart from the case regarding the exact position of the breakpoints (w = 0), which means that it underestimated the segment number and the estimated breakpoints were not located exactly at their true positions. Also CGHseg underestimated the number of segments because of low sensitivity and FDR, while HMM had low sensitivity and high FDR when w = 0 and vice versa in the other cases, which means that it often detected the true segment number, but it was unable to put the breakpoints at their exact position. Instead, BioHMM solved the issue of HMM with w = 0, but overall had a lower sensitivity than HMM. Rendersome missed several true aberrations (lowest sensitivity) and detected some false aberrations (medium FDR).

**Table 4:** Comparison among the piecewise constant methods on *Simulated Chromosomes*

**method**	**SSQ**	**MAD**	**accuracy**	**accuracy inside aberrations**	**accuracy outside aberrations**
mBPCR ${\hat{\rho }}^{2}$	1.70	0.00733	0.936	0.992	0.932
mBPCR ${\hat{\rho }}_{1}^{2}$	1.85	0.00781	0.929	0.993	0.920
CBS	1.56	0.00705	0.953	0.985	0.950
CGHseg	5.42	0.00795	0.925	0.885	0.956
HMM	4.47	0.00350	0.993	0.968	0.997
GLAD	4.15	0.00846	0.939	0.930	0.952
BioHMM	5.69	0.003647	0.990	0.949	0.999
Rendersome	19.13	0	0.920	0.289	1

The table shows the comparison of the level estimations obtained using several piecewise constant methods on dataset *Simulated Chromosomes*. In this comparison, the methods CBS and mBPCR exhibited the lowest SSQ error in the profile estimation and the highest accuracy inside the aberrated regions. On the other hand, HMM, BioHMM and Rendersome had the highest accuracy outside the aberrations, but a high SSQ error. Therefore, the former group of algorithms globally estimated a better profile than the latter. Because of its definition, the MAD error is less informative: it does not take into account if a small number of probes are wrongly estimated, but these probes could correspond to breakpoints or small aberrations.

#### Estimation with a continuous curve

**Figure 3:**
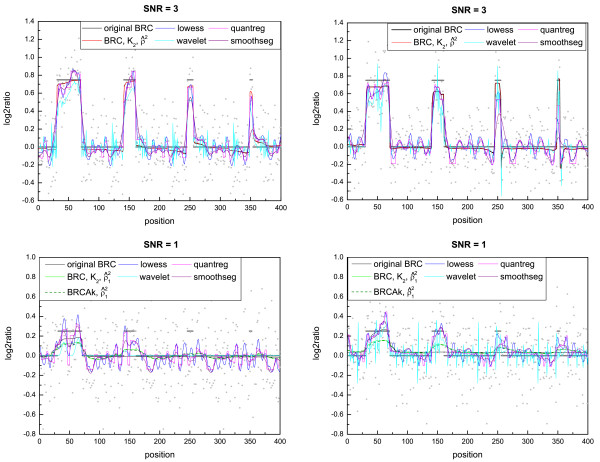
**Example of estimated regression curves**. The plots show the differences in the level estimation among the smoothing methods on samples with SNR = 3 and SNR = 1: some oscillate more in the regions outside the aberrations. In cases of high noise, the more oscillating the profiles are, the harder it is to identify which regions correspond to the aberrations. In each graph, the grey segments represent the true profile.

We compared the several versions of the Bayesian regression curves with methods which estimate the copy number as a continuous curve (lowess, wavelet, quantreg and smoothseg). Lowess is the acronym of "Locally Weighted Smoothing" (implemented in the stats library of R) and it is one of the methods considered in the comparison performed in [14]. As we saw previously, both the BRC, which uses ${\hat{K}}_{2}$, and the BRCAk perform well, so we tested both versions with both estimators of *ρ*^2^. Figure 3 shows examples of estimated profiles with these smoothing methods.

In general, we found that all methods detected the regions of aberration quite well (see, for example, Figures S.16 and S.18 in Additional file 2). The wavelet method showed a higher error in the level estimation of the aberrations in the datasets SNR = 3 and SNR = 1 (Figures S.16 and S.18 in Additional file 2). The methods lowess and quantreg had the highest RMSE in the collection *Cases*, while their error was not significantly different outside and inside the aberrations on datasets with SNR = 1, 3. Therefore, in the last cases the error was low inside the aberrations and high outside them in comparison with the other methods. The method smoothseg showed a similar behavior, but with a lower error.

Moreover, we found that the ROC measure was affected by oscillations in the estimated curve, which lead to ROC curves intersected and difficult to be interpreted (Figure S.15 in Additional file 2). This complex behavior is a consequence of the way in which lowess, wavelet, quantreg and smoothseg yielded oscillating curves with positive and negative values outside the aberrations; while BRCs estimated the true profile with a line almost flat and close to zero (see the examples in Figure 3).

In conclusion, the version of BRC with ${\hat{K}}_{2}$ and BRCAk gave in general a better estimation than the other BRCs and the other smoothing methods considered. Regarding the *ρ*^2 ^estimation, we found that it is better to use ${\hat{\rho }}^{2}$, if *σ*^2 ^<*ρ*^2^, and ${\hat{\rho }}_{1}^{2}$, if *σ*^2 ^> *ρ*^2^.

### Application to real data

In this subsection, we show how mBPCR performed compared to other piecewise constant estimation methods on the real data. We used samples from three mantle cell lymphoma cell lines (JEKO-1, GRANTA-519, REC-1) previously analyzed by us with the Affymetrix GeneChip Mapping 10K Array (Affymetrix, Santa Clara, CA), an oligonucleotides-based microarray [15]. We also used the data obtained on JEKO-1, by using the higher density Affymetrix GeneChip Mapping 250K Nsp Array. We considered eight recurrent gene regions of aberration in lymphoma plus other two gene regions (*BIRC3 *and *LAMP1*) and we compared the corresponding copy numbers obtained by the several piecewise constant methods with those obtained by the FISH technique in [15]. In the end, we also show a comparison among the estimated profiles of chromosome 11 of JEKO-1.

The 10K Array data used are freely available at the public repository Gene Expression Omnibus [16] with GEO accession: GSM95567, GSM95568 and GSM95570. The 250K Array data of cell line JEKO-1 will be soon available in the same repository.

With the current implementation, on a computer with dual CPU (AMD Opteron 250, 2.4 GHz) and 4 GB RAM, the algorithm needed about 4 minutes to analyze a 10K Array sample, while about 1 day to estimate the profile of a 250K Array sample. The computations were performed by chromosome (and by arm for the longest chromosomes in the 250K Array data) and using *k*_max _= 100.

#### Gene copy number estimation

To properly evaluate the methods, the knowledge of the true underling profile is required. In general, large aberrations on chromosomes can be detected with conventional karyotype analysis or with SKY-FISH and one could use this information for the evaluation procedure, but the width of these aberrations is so large that all the methods can detect them well, leading to a useless comparison. For this reason, we decided to take into account only genes to compare the piecewise constant methods.

In the comparison, as previously published [15], when two FISH copy numbers had been assigned to one gene, the first number should correspond to the copy number detected in the majority of the cells. We assigned two estimated copy numbers to one gene, when the position of the gene is between two SNPs and the method assigned two different values to these SNPs.

The results on REC-1 (Table S.3 in Additional file 2) did not show any significant difference among the methods, instead those on GRANTA-519 (Table S.4 in Additional file 2) showed that GLAD was unable to detect the true copy number in five cases, while HMM, BioHMM and Rendersome detected an amplification on *MALT1 *greater than what detected by FISH analysis. All methods did not detect the true copy number of *ATM*, probably because the SNPs around *ATM *are far away from the corresponding FISH region (about 1 Mb) and the deletion affects only this region. Only mBPCR with ${\hat{\rho }}_{1}^{2}$ and HMM detected a breakpoint between the two SNPs around *ATM *region, indicating a copy number change.

Regarding the JEKO-1 data, since the cell line is triploid, to obtain more realistic copy number value, we centered the estimated log_2_ratio around log_2 _3. With the denser 250K Array data, all methods behaved equally good. Only HMM had a problem in the detection of the breakpoint corresponding to the *C*-*MYC *amplification (see Table S.5 in Additional file 2). On both arrays, all methods identified a gain (copy number 3 or 4) at the *CCND1 *position, while the copy number detected by FISH is 2. This fact cannot be explained as previously for *ATM*, because this region is well covered by SNPs. Instead, on the JEKO-1 10K Array data (Table 5), the noisiest among all samples, we can see several cases in which CBS, HMM and GLAD did not detect correctly the gene copy number (for example, *BCL2 *and *MALT1*). This occurred more frequently to BioHMM and Rendersome, while only once to CGHseg (*LAMP1*). The method mBPCR with ${\hat{\rho }}_{1}^{2}$ always estimated gene copy numbers correctly, apart from *CCND1*.

**Table 5:** Copy number estimation results obtained on 10K Array data of sample JEKO-1

		**mBPCR**							
								
**gene region**	**FISH CN**	${\hat{\rho }}^{2}$	${\hat{\rho }}_{1}^{2}$	**CBS**	**CGHseg**	**HMM**	**GLAD**	**BioHMM**	**Rendersome**
*BCL6*	3/2	2.97	2.99	2.97	2.90	2.92	2.92	3.14	2.92
*C-MYC*	ampl	12.11	9.35	10.27	10.27	13.95	9.82	8.26	*13.10/3.11*
*CCND1*	2	*4.08*	*3.77*	*4.08*	*4.08*	*3.84*	*3.79*	*3.14*	*3.50*
*BIRC3*	4/5	4.08	4.29	4.08	4.08	3.84	3.79	*3.14*	3.50
*ATM*	4	4.08	4.29	4.08	4.08	3.84	3.79	*3.14*	*3.50/2.39*
*D13S319*	4	3.72	3.59	3.57	3.72	3.62	3.58	*3.14*	*3.43*
*LAMP1*	4	*3.41*	3.82	*3.41*	*3.41*	3.62	*2.49*	*3.14*	*3.43*
*TP53*	2/3	2.81	3.00	2.83	2.50	*3.52*	2.93	3.14	2.93
*MALT1*	4	3.63	3.62	*3.48*	3.64	*3.42*	*3.42*	*3.14*	*3.42*
*BCL2*	4	3.63	3.62	*3.48*	3.64	*3.42*	*3.42*	*3.14*	*3.42*

On this noisy data, BioHMM and Rendersome often estimated the gene copy number wrongly, while this occurred only sometimes to CBS, HMM and GLAD. The method mBPCR with ${\hat{\rho }}_{1}^{2}$ correctly estimated the gene copy numbers, apart from *CCND1 *whose copy number was estimated by all methods differently from the FISH technique.

#### Profile estimation

To compare the profile estimations, we chose the sample JEKO-1 because, using the results obtained on both types of array, we could at least understand which regions were more realistically estimated. For now, whole validated chromosomic profiles are not available. Among all chromosomes, we chose chromosome 11 since three of the previous genes belong to that: *CCND1 *(around 69.17 Mb), *BIRC3 *(around 101.7 Mb) and *ATM *(around 107.6 Mb).

**Figure 4:**
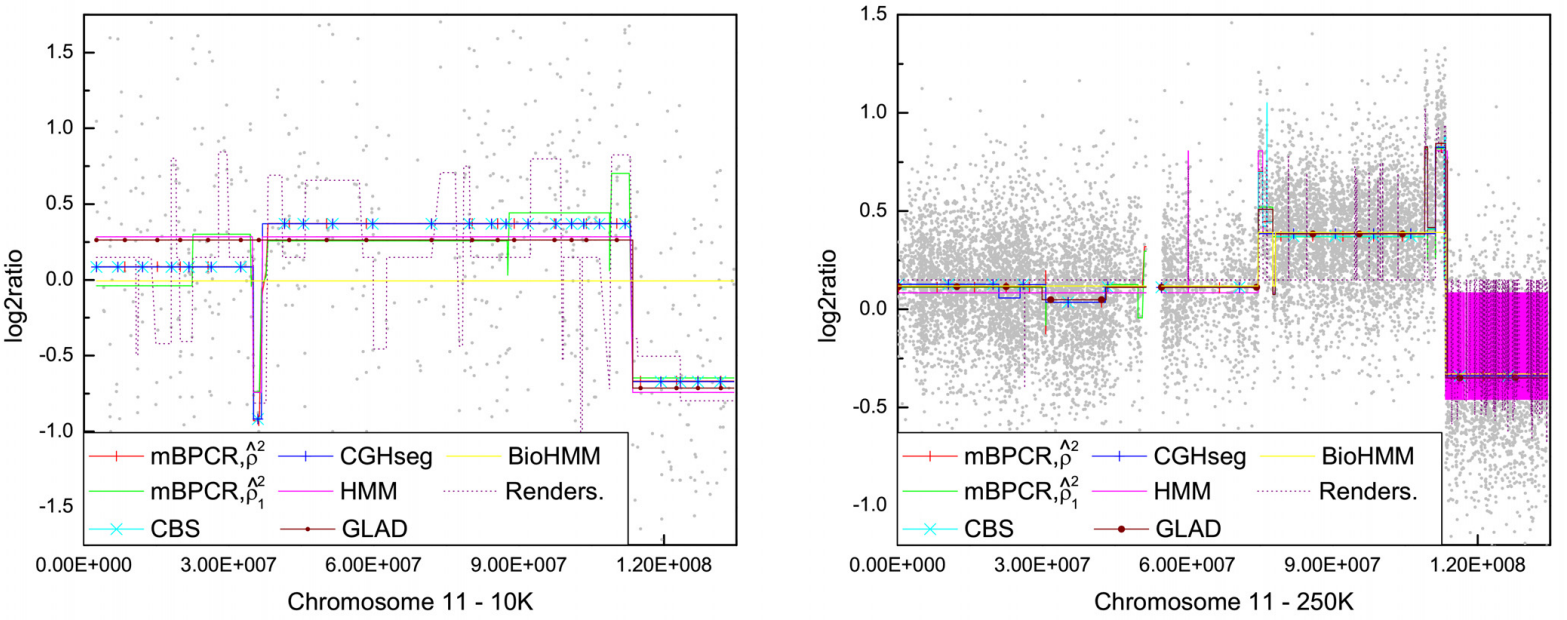
**Comparison among the estimated profiles of chromosome 11 of JEKO-1**. The figure shows the comparison among the piecewise constant estimated profiles of chromosome 11 of JEKO-1 using both 10K Array and 250K Array data. Only mBPCR with ${\hat{\rho }}_{1}^{2}$ was able to detect the high amplification after position 110 Mb on the 10K Array data. On the other hand, all methods (apart from BioHMM) recognized it on the 250K Array data.

From the graphs in Figure 4 we can observe that, among all the piecewise constant methods, only mBPCR with ${\hat{\rho }}_{1}^{2}$ was able to detect the high amplification after position 110 Mb on the 10K Array data, while it was recognized by all methods (apart from BioHMM) on the 250K Array data. Moreover, on the 10K Array data, almost all methods detected a false deletion around position 3 Mb, due to the presence of a sequence of outliers, and BioHMM did not find any copy number change in the chromosome. On the 250K Array data, HMM and Rendersome had problems in recognizing the last part of the chromosome as a flat region. Moreover, on the 10K Array data, Rendersome estimated several outliers as true aberrations and, on the 250K Array data, it was unable (contrary to all other algorithms) to identify the whole region from about 78 Mb to 111 Mb as gained.

## Conclusion

We introduced new estimators for the parameters involved in BPCR and we selected the best ones on the basis of theoretic and empirical results. In particular, we found that the best way is to estimate the segment number with ${\hat{K}}_{2}$ and the boundaries with ${\hat{T}}_{BinErrAk}$ (or possibly ${\hat{T}}_{joint}$). We call mBPCR the BPCR version which uses ${\hat{K}}_{2}$ and ${\hat{T}}_{BinErrAk}$.

Concerning the estimation of the variance of the segment levels, we found that the original estimator ${\hat{\rho }}^{2}$ overestimates *ρ*^2 ^(variance of the segment levels) by an addendum proportional to *σ*^2 ^(variance of the noise), see Equation (31). Hence, the estimation fails when *σ*^2 ^> *ρ*^2^. The new estimator ${\hat{\rho }}_{1}^{2}$ is more precise but slightly underestimates *ρ*^2^, leading to an overestimation of the segment number. Applying both estimators on artificial datasets, we found that, in general, the best way is to use ${\hat{\rho }}^{2}$ when *σ*^2 ^<*ρ*^2 ^(low noise), but to use ${\hat{\rho }}_{1}^{2}$ when *σ*^2 ^> *ρ*^2 ^(high noise), even if it could lead to a slight overfitting. On real DNA copy number data, commonly *σ*^2 ^> *ρ*^2^.

We also compared mBPCR with other methods which also estimate the copy number as a piecewise constant function: CBS, HMM, CGHseg, GLAD, BioHMM and Rendersome. As a whole, the results showed that mBPCR gave the best estimation on the dataset used. However, when *σ*^2 ^≫ *ρ*^2 ^it is hard to understand which method is the most appropriate. Most of the other methods were not able to detect aberrations with a small width (5 and 10 probes) and the same was true for mBPCR using ${\hat{\rho }}^{2}$. On the other hand, the use of ${\hat{\rho }}_{1}^{2}$ led to the detection of the smaller segments, but the larger ones were often divided in small segments and sometimes the segments consisted of only one point. The optimal choice of the *ρ*^2 ^estimator is still not fully determined.

The new estimators improved also BRC, which is a Bayesian regression with a smooth curve. Moreover, we derived a formula to estimate BRC without employing the estimated number of segments. We referred to it as BRCAk. Applying these methods to artificial data, the best estimators were found to be the BRC version with ${\hat{K}}_{2}$ and BRCAk. About the choice of the two estimators of *ρ*^2^, we found a similar conclusion as before, with the advantage of being less problematic when *σ*^2 ^≫ *ρ*^2^. We compared these two regression methods with other methods which estimate the copy number data as a continuous curve: wavelet, lowess, quantreg and smoothseg. The results showed that our modified regression methods were the most appropriate for the estimation of the segment levels on the datasets considered.

Even though these smoothing methods seem to have less problems in the estimation and the error measures (for example, the ROC curve) suggest that their estimation is even better than the piecewise constant estimation, these methods do not detect the position of the breakpoints explicitly and hence the changes in the value of the segment level. Thus, they seem to be less adequate in practice.

For this reason, we feel that the ROC curve cannot be used as the only measure to compare methods, as previously done in [14]. The RMSE is generally an acceptable measure, but we observed that in some cases even this is not sufficient, due to the overfitting. Willenbrock and Fridlyand [13] proposed other measures to compare methods for copy number estimation, regarding both breakpoint and level estimation. In particular, the sensitivity measure of breakpoint estimation is useful to select which methods should be used, because it quantifies the precision of the methods in determining the position of the breakpoints.

Finally, we have applied mBPCR and all other piecewise constant regression methods to real data. The comparisons showed that mBPCR estimated well the copy number of the genes. On these data, in most cases the choice of the *ρ*^2 ^estimator did not affect the analysis.

In comparison with the other methods, the current implementation of our algorithm is computationally intensive. The real computational time can be reduced linearly diminishing *k*_max _and quadratically diminishing the number of probes. Moreover, the computation can be easily parallelized by arm and by chromosome, reducing further the calculation time.

In cancer research, the accuracy in the DNA copy number estimation is crucial for the correct determination of the mutations that characterize the disease. In particular, the estimation of the breakpoints must be precise to detect correctly which genes are affected by these genomic aberrations. As recently shown [17], SNP microarrays can also potentially detect the breakpoints involved in unbalanced translocations, allowing the identification of fusion genes (i.e. hybrid genes created by joining portions of two different genes). In this context, the use of our method can highly improve the disease investigation, because it accurately determines breakpoints, is less sensitive to high noise and generally outperforms all the methods considered in our comparisons. Moreover, smoothing algorithms are clearly not suitable for such analysis.

## Availability and requirements

**Project name**: mBPCR.

**Project home page**: .

**Operating systems**: the software should run in Linux, Mac-OS or Windows. Tests were performed on Windows and Linux systems.

**Programming language**: R.

**Other requirements**: none.

**Licence**: GNU GPL.

**Any restrictions to use by non-academics**: none.

## Authors' contributions

PMVR carried out the study and wrote the manuscript. MH and IK supervised the statistical analysis. FB supervised the validation study and provided the real data. All authors read and approved the final manuscript.

## Supplementary Material

Additional file 1 **mBPCR source code.** This zipped file contains the source code of the mBPCR algorithm in R, including help files, sample data and examples. Click here for file

Additional file 2 **Supplementary material.** This file contains a brief description of the dynamic program used to implement the method, a formal definition of some error measure used, some supplementary tables and some supplementary figures. Click here for file
