# Supplementary material for: Bayesian DNA copy number analysis
Source: BMC Bioinformatics. 2009 Jan 8;10:10. doi: 10.1186/1471-2105-10-10 (PMC2674052; doi:10.1186/1471-2105-10-10)
Supplement: Additional file 1 — mBPCR source code. This zipped file contains the source code of the mBPCR algorithm in R, including help files, sample data and examples. [file 1471-2105-10-10-S1.zip › mBPCRsource_code/html/estGlobParam.html]

R: Estimate global parameters of copy number data

|  |  |
| --- | --- |
| estGlobParam {mBPCR} | R Documentation |

## Estimate global parameters of copy number data

### Description

Function to estimate the global parameters of copy number data: the mean and the variance of the segment levels (called `nu` and `rhoSquare`, respectively), the variance of the noise (`sigmaSquare`). It is possible
to choose the estimator of `rhoSquare` (i.e. either \hat{rho}\_1^2 or \hat{rho}^2) and by default \hat{rho}\_1^2 is used.

### Usage

```
  estGlobParam(y, nu=NULL, rhoSquare=NULL, sigmaSquare=NULL, typeEstRho=1)
```

### Arguments

|  |  |
| --- | --- |
| `y` | array containing the log2ratio of the copy number data |
| `nu` | mean of the segment levels. If `nu=NULL`, then the algorithm estimates it on the sample. |
| `rhoSquare` | variance of the segment levels. If `rhoSquare=NULL`, then the algorithm estimates it on the sample. |
| `sigmaSquare` | variance of the noise. If `sigmaSquare=NULL`, then the algorithm estimates it on the sample. |
| `typeEstRho` | choice of the estimator of `rhoSquare`. If `typeEstRho=1`, then the algorithm estimates `rhoSquare` with \hat{rho}\_1^2, if `typeEstRho=0` estimates it with \hat{rho}^2. |

### Value

A list cointaining `nu`, `rhoSquare` and `sigmaSquare`.

### Examples

```
##import the 10K data of cell line REC  
##for windows
path <- 'data\\rec10k.dat'
##for linux
##path <- 'data//rec10k.dat'
rec10k <- importCNData(path, NRowSkip=1)
##estimation of all the global parameters (the variance of the segment is estimated with \hat{rho}^2_1)
estGlobParam(rec10k$logratio)

```
---


[Package mBPCR version 1.0 Index]
```
```
