# Supplementary material for: Bayesian DNA copy number analysis
Source: BMC Bioinformatics. 2009 Jan 8;10:10. doi: 10.1186/1471-2105-10-10 (PMC2674052; doi:10.1186/1471-2105-10-10)
Supplement: Additional file 1 — mBPCR source code. This zipped file contains the source code of the mBPCR algorithm in R, including help files, sample data and examples. [file 1471-2105-10-10-S1.zip › mBPCRsource_code/html/00Index.html]

R: mBPCR

# mBPCR for DNA copy number data analysis

---

## Documentation for package `mBPCR' version 1.0


## Help Pages

|  |  |
| --- | --- |
| computeMBPCR | Estimate the copy number profile |
| estGlobParam | Estimate global parameters of copy number data |
| estProfileWithMBPCR | Estimate and print the copy number profile of some chromosomes of a sample |
| importCNData | Import the copy number data |
| logAdd | Overflow-safe computation of the logarithm of a sum |
| plotEstProfile | Plot the estimated profile of copy number data |
| printEstProfile | Print the estimated profile of copy number data |
| mBPCR-internal | Internal mBPCR functions |
