# Supplementary material for: Bayesian DNA copy number analysis
Source: BMC Bioinformatics. 2009 Jan 8;10:10. doi: 10.1186/1471-2105-10-10 (PMC2674052; doi:10.1186/1471-2105-10-10)
Supplement: Additional file 1 — mBPCR source code. This zipped file contains the source code of the mBPCR algorithm in R, including help files, sample data and examples. [file 1471-2105-10-10-S1.zip › mBPCRsource_code/html/plotEstProfile.html]

R: Plot the estimated profile of copy number data

|  |  |
| --- | --- |
| plotEstProfile {mBPCR} | R Documentation |

## Plot the estimated profile of copy number data

### Description

Function to plot the estimated profiles of copy number data.
The function gives also the possibility to print each graph on a file.

### Usage

```
  plotEstProfile(path='', sampleName='', chr, position, logratio, chrToBePlotted, estPC, maxProbeNumber,
                 legendPosition='bottomleft', regrCurve=NULL, regr=NULL)
```

### Arguments

|  |  |
| --- | --- |
| `path` | path of the folder where the user wants to print the plots of the estimated profiles (it must end with '\\' in windows, or '//' in linux). If path='', they will be printed in the working directory, while if `path=NULL`, the plots will not be printed. |
| `sampleName` | name of the sample, if the user wants to put it in the name of the files printed |
| `snpName` | array containing the name of each probe |
| `chr` | array containing the name of the chromosome to which each probe belongs |
| `position` | array containing the physical position of each probe |
| `logratio` | array containing the log2ratio of the raw copy number data |
| `chrToBePlotted` | array containing the name of the estimated chromosomes, that the user wants to plot. The possible values of the chromosomes are: an integer from 1 to 22 and 'X'. |
| `estPC` | array containing the estimated copy number profile as a piecewise constant function. If `estPC=NULL`, only the estimated Bayesian regression curve is plotted. |
| `maxProbeNumber` | maximum number of probes that a chromosome (or arm of a chromosome) can have to be analyzed. The procedure of profile estimation needs the computation of an array of length `(length(chromosome)+1)*(length(chromosome)+2)/2`. To be sure to have set this parameter correctly, try to create the array `A <- array(1, dim=(maxProbeNumber+1)*(maxProbeNumber+2)/2)`, before starting with the estimation procedure. |
| `legendPosition` | string containing the position of the legend in the plot. The possible values are the same used in the function `plot`. |
| `regrCurve` | array containing the estimated regression curve. If `regrCurve=NULL`, then the estimated Bayesian regression curve is not plotted. If `regrCurve!=NULL` and also `estPC!=NULL` both estimated profiles are plotted on the same graph. |
| `regr` | choice of the computation of the regression curve. If `regr=NULL`, then the regression curve was not computed (then the estimated Bayesian regression curve is not plotte), if `regr=1` the Bayesian Regression Curve was computed (mBRC with K\_2), if `regr=2` the Bayesian Regression Curve Averaging over k was computed (BRCAk). |

### Value

The function plots the estimated profiles of the chromosomes of `chrToBePlotted`, separately. Eventually, the function prints the same plots in .eps format.

### Examples

```
##import the 10K data of cell line REC
##for windows
path <- 'data\\rec10k.dat'
##for linux
##path <- 'data//rec10k.dat'
rec10k <- importCNData(path, NRowSkip=1)
##estimation of chromosomes 2, 3 and 4
results <- estProfileWithMBPCR(path='', sampleName='rec10k', rec10k$snpName, rec10k$chr, rec10k$position, rec10k$logratio, chrToBeAnalyzed=2:4, maxProbeNumber=2000)
##plot the corresponding estimated profiles (the graphs are also printed in the working directory)
plotEstProfile(path='', sampleName='rec10k', rec10k$chr, rec10k$position, rec10k$logratio, chrToBePlotted=2:4, results$estPC, maxProbeNumber=2000)

```
---


[Package mBPCR version 1.0 Index]
```
```
