# Supplementary material for: Bayesian DNA copy number analysis
Source: BMC Bioinformatics. 2009 Jan 8;10:10. doi: 10.1186/1471-2105-10-10 (PMC2674052; doi:10.1186/1471-2105-10-10)
Supplement: Additional file 1 — mBPCR source code. This zipped file contains the source code of the mBPCR algorithm in R, including help files, sample data and examples. [file 1471-2105-10-10-S1.zip › mBPCRsource_code/html/computeMBPCR.html]

R: Estimate the copy number profile

|  |  |
| --- | --- |
| computeMBPCR {mBPCR} | R Documentation |

## Estimate the copy number profile

### Description

Function to estimate the copy number profile with a piecewise constant function using mBPCR. Eventually, it is possible to estimate the profile with a
smoothing curve using either the Bayesian Regression Curve with K\_2 (BRC with K\_2) or the Bayesian Regression Curve Averaging over k (BRCAk). It is also possible
to choose the estimator of rhoSquare (i.e. either \hat{rho}\_1^2 or \hat{rho}^2) and by default \hat{rho}\_1^2 is used.

### Usage

```
  computeMBPCR(y, kMax=50, nu=NULL, rhoSquare=NULL, sigmaSquare=NULL, typeEstRho=1, regr=NULL)
```

### Arguments

|  |  |
| --- | --- |
| `y` | array containing the log2ratio of the copy number data |
| `kMax` | maximum number of segments |
| `nu` | mean of the segment levels. If `nu=NULL`, then the algorithm estimates it on the sample. |
| `rhoSquare` | variance of the segment levels. If `rhoSquare=NULL`, then the algorithm estimates it on the sample. |
| `sigmaSquare` | variance of the noise. If `sigmaSquare=NULL`, then the algorithm estimates it on the sample. |
| `typeEstRho` | choice of the estimator of `rhoSquare`. If `typeEstRho=1`, then the algorithm estimates `rhoSquare` with \hat{rho}\_1^2, if `typeEstRho=0` estimates it with \hat{rho}^2. |
| `regr` | choice of the computation of the regression curve. If `regr=NULL`, then the regression curve is not computed, if `regr=1` the Bayesian Regression Curve with K\_2 is computed (BRC with K\_2), if `regr=2` the Bayesian Regression Curve Averaging over k is computed (BRCAk). |

### Value

A list cointaining: `estK` (the estimated number of segments), `estBoundaries` (the estimated boundaries),
`estPC` (the estimated profile with mBPCR), `nu`, `rhoSquare`, `sigmaSquare`,
`postProbT` (i.e. for each probe, the posterior probablity to be a breakpoint) and, eventually, `regrCurve`
(i.e. the estimated bayesian regression curve).

### Examples

```
##import the 250K NSP data of chromosome 11 of cell line JEKO-1 
##for windows 
path <- 'data\\jekoChr11Array250Knsp.dat'
##for linux
##path <- 'data//jekoChr11Array250Knsp.dat'
jekoChr11Array250Knsp <- importCNData(path, NRowSkip=1)

##first example 
## we select a part of chromosome 11
y <- jekoChr11Array250Knsp$logratio[10600:11600]
p <- jekoChr11Array250Knsp$position[10600:11600]
##we estimate the profile using the global parameters estimated on the whole genome
results <- computeMBPCR(y, nu=-3.012772e-10, rhoSquare=0.0479, sigmaSquare=0.0699, regr=2)
plot(p,y)
points(p, results$estPC, type='l', col='red')
points(p, results$regrCurve, type='l', col='green')

##second example 
## we select a part of chromosome 11
y <- jekoChr11Array250Knsp$logratio[6400:6900]
p <- jekoChr11Array250Knsp$position[6400:6900]
##we estimate the profile using the global parameters estimated on the whole genome
results <- computeMBPCR(y, nu=-3.012772e-10, rhoSquare=0.0479, sigmaSquare=0.0699, regr=1)
plot(p, y)
points(p, results$estPC, type='l', col='red')
points(p, results$regrCurve,type='l', col='green')

```
---


[Package mBPCR version 1.0 Index]
```
```
