# Supplementary material for: Bayesian DNA copy number analysis
Source: BMC Bioinformatics. 2009 Jan 8;10:10. doi: 10.1186/1471-2105-10-10 (PMC2674052; doi:10.1186/1471-2105-10-10)
Supplement: Additional file 1 — mBPCR source code. This zipped file contains the source code of the mBPCR algorithm in R, including help files, sample data and examples. [file 1471-2105-10-10-S1.zip › mBPCRsource_code/html/mBPCR-internal.html]

R: Internal mBPCR functions

|  |  |
| --- | --- |
| mBPCR-internal {mBPCR} | R Documentation |

## Internal mBPCR functions

### Description

Internal functions of package mBPCR.

### Usage

```
computeA10(i, j, y, mglob, varglob, varseg)
computeLA0Vect(y, mglob, varglob, varseg)
computePCReg(y, lA0, lL, lR, mglob, varglob, varseg, kMax=50, regr=NULLindexLA0(r, c, n)
computeRecursions(lA0, n, kMax=50)
computeRegrCurve(y, typeRegr=1, n, kMax=50, lL, lR, lA0, mglob, varglob, varseg, option)
indexLA0(r, c, n)
```

### Details

These functions are not to be called directly by the user

---

[Package *mBPCR* version 1.0 Index]
