# Supplementary material for: Bayesian DNA copy number analysis
Source: BMC Bioinformatics. 2009 Jan 8;10:10. doi: 10.1186/1471-2105-10-10 (PMC2674052; doi:10.1186/1471-2105-10-10)
Supplement: Additional file 1 — mBPCR source code. This zipped file contains the source code of the mBPCR algorithm in R, including help files, sample data and examples. [file 1471-2105-10-10-S1.zip › mBPCRsource_code/html/printEstProfile.html]

R: Print the estimated profile of copy number data

|  |  |
| --- | --- |
| printEstProfile {mBPCR} | R Documentation |

## Print the estimated profile of copy number data

### Description

Function to print the results of the profile estimation of copy number data

### Usage

```
  printEstProfile(path='', sampleName='', snpName, chr, position, logratio,
                  chrToBePrinted, estPC, estBoundaries=NULL, postProbT=NULL,
                  regrCurve=NULL, regr=NULL)
```

### Arguments

|  |  |
| --- | --- |
| `path` | path of the folder where the user wants to print the results of the estimation (it must end with '\\' in windows, or '//' in linux). If path='', they will be printed in the working directory |
| `sampleName` | name of the sample. If the name of the sample if provided, it is used to named the printed files. |
| `snpName` | array containing the name of each probe |
| `chr` | array containing the name of the chromosome to which each probe belongs |
| `position` | array containing the physical position of each probe |
| `logratio` | array containing the log2ratio of the raw copy number data |
| `chrToBePrinted` | array containing the name of the estimated chromosomes, that the user wants to print. The possible values of the chromosomes are: an integer from 1 to 22 and 'X'. |
| `estPC` | array containing the estimated copy number profile as a piecewise constant function |
| `estBoundaries` | list containing the vectors of the estimated breakpoints, for each of the chromosomes mentioned in `chrToBePrinted`. If `estBoundaries=NULL`, then this information is not printed. |
| `postProbT` | list containing the vectors of the posterior probabilities to be a breakpoint of the estimated breakpoints, for each of the chromosomes mentioned in `chrToBePrinted`. If `postProbT=NULL`, then this information is not printed in the file containing the estimated breakpoints. |
| `regrCurve` | array containing the estimated regression curve. If `regrCurve=NULL`, then the file containing this information is not printed. |
| `regr` | choice of the computation of the regression curve. If `regr=NULL`, then the regression curve was not computed (then the file containing this information is not printed), if regr=1 the Bayesian Regression Curve with K\_2 was computed (BRC with K\_2), if regr=2 the Bayesian Regression Curve Averaging over k was computed (BRCAk). |

### Value

The function print at maximum three files:

- one containing the estimated profile with mBPCR (the columns are: 'SNP\_name', 'chromosome', 'position', 'rawLog2ratio', 'mBPCR\_estimate')
- one containing a summary about the estimated profile with mBPCR (the columns are: 'SNP\_name(start)', 'SNP\_name(end)', 'chromosome', 'position(start)',
  'position(end)', 'n\_probes', 'mBPCR\_estimate' and, eventually, 'breakpointPostProb'). This file is not printed if `estBoundaries=NULL`.
- one containing the estimated profile with a regression curve (the columns are: 'SNP\_name', 'chromosome', 'position', 'rawLog2ratio' and the name of the
  regression curve used). This file is not printed if `regrCurve=NULL`.

### Examples

```
##import the 10K data of cell line REC
##for windows
path <- 'data\\rec10k.dat'
##for linux
##path <- 'data//rec10k.dat'
rec10k <- importCNData(path, NRowSkip=1)
#estimation of the global parameters
param <- estGlobParam(rec10k$logratio)
##estimation of chromosome 3
results=computeMBPCR(rec10k$logratio[which(rec10k$chr == 3)], nu=param$nu, rhoSquare=param$rhoSquare, sigmaSquare=param$sigmaSquare)
##print the estimated profile of chromosome 3
estPC <- array(dim=length(rec10k$snpName))
estBoundaries <- list(dim=1)
postProbT <- list(dim=1)
estPC[rec10k$chr == 3] <- results$estPC
estBoundaries[[1]] <- results$estBoundaries
postProbT[[1]] <- c(results$postProbT[results$estBoundaries[-results$estK]],1)
printEstProfile(path='', sampleName='rec10k', rec10k$snpName, rec10k$chr, rec10k$position, rec10k$logratio, chrToBePrinted=3, estPC, estBoundaries, postProbT)

```
---


[Package mBPCR version 1.0 Index]
```
```
