# Supplementary material for: Bayesian DNA copy number analysis
Source: BMC Bioinformatics. 2009 Jan 8;10:10. doi: 10.1186/1471-2105-10-10 (PMC2674052; doi:10.1186/1471-2105-10-10)
Supplement: Additional file 1 — mBPCR source code. This zipped file contains the source code of the mBPCR algorithm in R, including help files, sample data and examples. [file 1471-2105-10-10-S1.zip › mBPCRsource_code/html/estProfileWithMBPCR.html]

R: Estimate and print the copy number profile of some chromosomes of a sample

|  |  |
| --- | --- |
| estProfileWithMBPCR {mBPCR} | R Documentation |

## Estimate and print the copy number profile of some chromosomes of a sample

### Description

Function to estimate the copy number profile with a piecewise constant function using mBPCR. Eventually, it is possible to estimate the profile with a
smoothing curve, using either the Bayesian Regression Curve with K\_2 (BRC with K\_2) or the Bayesian Regression Curve Averaging over k (BRCAk). It is also possible
to choose the estimator of `rhoSquare` (i.e. either \hat{rho}\_1^2 or \hat{rho}^2) and by default \hat{rho}\_1^2 is used.
The function gives also the possibility to print the results.

### Usage

```
  estProfileWithMBPCR(path='', sampleName='', snpName, chr, position, logratio, chrToBeAnalyzed, maxProbeNumber, 
                      rhoSquare=NULL, kMax=50, nu=NULL, sigmaSquare=NULL, typeEstRho=1, regr=NULL)
```

### Arguments

|  |  |
| --- | --- |
| `path` | pathway of the folder where the user wants to print the results of the estimation (it must end with '\\' in windows, or '//' in linux). By default the results are printed in the working directory, while if `path=NULL` the results are not printed. |
| `sampleName` | name of the sample analyzed. If the name of the sample if provided, it is used to named the printed files. |
| `snpName` | array containing the name of each probe |
| `chr` | array containing the name of the chromosome to which each of the probes belongs |
| `position` | array containing the physical position of each probe |
| `logratio` | array containing the log2ratio of the raw copy number data |
| `chrToBeAnalyzed` | array containing the name of the chromosomes that the user wants to analyze. The possible values of the chromosomes are: an integer from 1 to 22 and 'X'. |
| `maxProbeNumber` | maximum number of probes that a chromosome (or arm of a chromosome) can have to be analyzed. The procedure of profile estimation needs the computation of an array of length `(length(chromosome)+1)*(length(chromosome)+2)/2`. To be sure to have set this parameter correctly, try to create the array `A <- array(1, dim=(maxProbeNumber+1)*(maxProbeNumber+2)/2)`, before starting with the estimation procedure. |
| `rhoSquare` | variance of the segment levels. If `rhoSquare=NULL`, then the algorithm estimates it on the sample. |
| `kMax` | maximum number of segments |
| `nu` | mean of the segment levels. If `nu=NULL`, then the algorithm estimates it on the sample. |
| `sigmaSquare` | variance of the noise. If `sigmaSquare=NULL`, then the algorithm estimates it on the sample. |
| `typeEstRho` | choice of the estimator of `rhoSquare`. If `typeEstRho=1`, then the algorithm estimates `rhoSquare` with \hat{rho}\_1^2, if `typeEstRho=0` estimates it with \hat{rho}^2. |
| `regr` | choice of the computation of the regression curve. If `regr=NULL`, then the regression curve is not computed, if `regr=1` the Bayesian Regression Curve is computed (BRC with K\_2), if `regr=2` the Bayesian Regression Curve Averaging over k is computed (BRCAk). |

### Value

A list cointaining: `estPC` (i.e. an array containing the estimated profile with mBPCR), `estBoundaries` (i.e. the list of estimated breakpoints for each of the analyzed chomosomes),
`postProbT` (i.e. the list of the posterior probablity to be a breakpoint for each estimated breakpoint of the analyzed chomosomes) and, eventually, `regrCurve`
(i.e. an array containing the estimated bayesian regression curve). `estPC` and `regrCurve` have the same length of `logratio`, hence their components
corresponding to the not analyzed chromosomes are equal to `NA`.

### Examples

```
##import the 10K data of cell line REC  
##for windows
path <- 'data\\rec10k.dat'
##for linux
##path <- 'data//rec10k.dat'
rec10k <- importCNData(path, NRowSkip=1)
##estimation of the profile of all chromosomes
results=estProfileWithMBPCR(path='', sampleName='rec10k', rec10k$snpName, rec10k$chr, rec10k$position, rec10k$logratio, chrToBeAnalyzed=c(1:22,'X'), maxProbeNumber=2000)
##plot the estimated profile of chromosome 3
y <- rec10k$logratio[rec10k$chr == 3]
p <- rec10k$position[rec10k$chr == 3]
plot(p, y)
points(p, results$estPC[rec10k$chr == 3], type='l', col='red')

```
---


[Package mBPCR version 1.0 Index]
```
```
