# Supplementary material for: Bayesian DNA copy number analysis
Source: BMC Bioinformatics. 2009 Jan 8;10:10. doi: 10.1186/1471-2105-10-10 (PMC2674052; doi:10.1186/1471-2105-10-10)
Supplement: Additional file 1 — mBPCR source code. This zipped file contains the source code of the mBPCR algorithm in R, including help files, sample data and examples. [file 1471-2105-10-10-S1.zip › mBPCRsource_code/html/importCNData.html]

R: Import the copy number data

|  |  |
| --- | --- |
| importCNData {mBPCR} | R Documentation |

## Import the copy number data

### Description

Function to import the raw copy number data.

### Usage

```
  importCNData(path, NRowSkip, ifLogRatio=1)
```

### Arguments

|  |  |
| --- | --- |
| `path` | path of the file containing the copy number data. The file must contain a table, where in the first column there are the names of the probes (snpName), in the second one, the chromosome to which each probe belongs, in the third one, the phisical positions of the probes and in the forth one, the copy number data. |
| `NRowSkip` | number of row to skip in the file, before the table. The names of the columns are to be skipped. |
| `ifLogRatio` | denotes if the data are either the log2ratio of raw copy number data or raw copy number data. By default, they are considered as log2ratio data, otherwise (`ifLogRatio=0`) they are transformed in log2ratio data. |

### Value

A list cointaining: `snpName` (i.e. an array containing the names of the probes), `chr` (i.e. an
array containing the name of the chromosome to which each probe belongs), `position` (i.e. an array
containing the physical position of each probe) and `logratio` (i.e. an array containing the log2ratio of the raw copy number data).

### Examples

```
##import the 10K data of cell line REC
##for windows
path <- 'data\\rec10k.dat'
##for linux
##path <- 'data//rec10k.dat'
rec10k <- importCNData(path, NRowSkip=1)
plot(rec10k$position[rec10k$chr == 3], rec10k$logratio[rec10k$chr == 3])

```
---


[Package mBPCR version 1.0 Index]
```
```
