# Supplementary material for: Bayesian DNA copy number analysis
Source: BMC Bioinformatics. 2009 Jan 8;10:10. doi: 10.1186/1471-2105-10-10 (PMC2674052; doi:10.1186/1471-2105-10-10)
Supplement: Additional file 2 — Supplementary material. This file contains a brief description of the dynamic program used to implement the method, a formal definition of some error measure used, some supplementary tables and some supplementary figures. [file 1471-2105-10-10-S2.pdf]

Supplementary material

S.1 The dynamic program

The Bayesian estimation needs the computation of the posterior probabilities of the variables involved, but the complexity of the problem does not allow us to find them analytically. Fortunately the computation can be done efficiently using dynamic programming. In this section we will briefly present the algorithm, while a more detailed explanation can be found in Hutter [11,12].

First of all, we introduce some notations:

$$\begin{aligned} \mathbf{M}_{l,m} &= (\mathrm{M}_{l+1},\ \ldots,\ \mathrm{M}_m) \\ \boldsymbol{T}_{l,m} &= (T_l,\ \ldots,\ T_m) \\ \boldsymbol{Y}_{i,j} &= (Y_{i+1},\ \ldots,\ Y_j) \\ K_{i,j} &= \text{number of the segments in } \boldsymbol{Y}_{i,j}. \end{aligned}$$

The dynamic program is based on the fact that, for a fixed segment number and partition, the data points that belong to different segments are independent. Then, the joint probability distribution of the data points and the levels in $m-l$ segments can be decomposed in the product of the same joint probability distribution in the first $p-l$ segments and in the last $m-p$ segments, for any p such that $l < p \leq m$:

$$\begin{aligned} & f(\boldsymbol{y}_{t_l,t_m},\, \boldsymbol{\mu}_{l,m} \,|\, \boldsymbol{t}_{l,m},\, K_{t_l,t_m} = m-l) \\ &= \prod_{i=l}^{m-1} f(\boldsymbol{y}_{t_i,t_{i+1}} \,|\, \mu_{i+1}, \boldsymbol{t}_{i,i+1}, K_{t_i,t_{i+1}} = 1) f(\mu_{i+1} \,|\, \boldsymbol{t}_{i,i+1}, K_{t_i,t_{i+1}} = 1) \\ &= f(\boldsymbol{y}_{t_l,t_p}, \boldsymbol{\mu}_{l,p} \,|\, \boldsymbol{t}_{l,p}, K_{t_l,t_p} = p-l) f(\boldsymbol{y}_{t_p,t_m}, \boldsymbol{\mu}_{p,m} \,|\, \boldsymbol{t}_{p,m}, K_{t_p,t_m} = m-p). \end{aligned} \tag{S.1}$$

We define the following quantity to perform the dynamic program

$$\mathrm{A}^r_{i,j} := \int_{\mathbb{R}} \mu^r_m f(\boldsymbol{y}_{i,j}, \mu_m |\, K_{i,j} = 1) d\mu_m \qquad \text{for all } i < m \leq j, \tag{S.2}$$

where $\widetilde{\mu}_{i+1} = \ldots = \widetilde{\mu}_j = \mu_m$, since all the data points considered belong to the same segment, which we call m. One should notice that, if $r = 0$,

$$\mathrm{A}^0_{i,j} = f(\boldsymbol{y}_{i,j} \,|\, K_{i,j} = 1), \tag{S.3}$$

i.e., it is the density of the data $\boldsymbol{y}_{i,j}$ given that they belong to the same segment, while if $r \geq 1$, $\mathrm{A}^r_{i,j}$ is related to the moments of M_m with respect to the conditional probability given $\boldsymbol{y}$, $\boldsymbol{t}$ and k,

$$E[\mathrm{M}^r_m \,|\, \boldsymbol{y}, \boldsymbol{t}, k] \;=\; E[\mathrm{M}^r_m \,|\, \boldsymbol{y}_{T_{m-1},T_m}, T_{m-1} = i, T_m = j, K_{T_{m-1},T_m} = 1] \;=\; \frac{\mathrm{A}^r_{i,j}}{\mathrm{A}^0_{i,j}} \tag{S.4}$$

where $i = t_{m-1}$ and $j = t_m$, for all $m = 1,\ \ldots,\ k$.

The left and right recursion of the dynamic program are made on the following quantities

$$\mathrm{L}_{k+1,j} \;:=\; \binom{j-1}{k} f(\boldsymbol{y}_{0,j}|\, K_{0,j} = k+1) \tag{S.5}$$

$$\mathrm{R}_{k+1,i} \;:=\; \binom{n-i-1}{k} f(\boldsymbol{y}_{i,n}|\, K_{i,n} = k+1). \tag{S.6}$$

Given the position of t_k, the probability distribution of the data in $k+1$ segments can be decomposed in the product between the joint distribution in the first k segments and the joint distribution in the last segment. Hence, we can compute the probability distribution of the data in $k+1$ segments summing this products for all possible positions of t_k,

$$\mathrm{L}_{k+1,j} \;=\; \sum_{h=k}^{j-1} \mathrm{L}_{k,h}\mathrm{A}^0_{h,j}. \tag{S.7}$$

Similarly, we can sum, over all possible position of t_1, the products between the joint distribution in the first segment and the joint distribution in the last k segments, obtaining

$$\mathrm{R}_{k+1,i} \;=\; \sum_{h=i+1}^{n-k} \mathrm{A}^0_{i,h}\mathrm{R}_{k,h}. \tag{S.8}$$

The recursion starts with $\mathrm{L}_{0,j} := \delta_{0,j}$ and $\mathrm{R}_{0,i} := \delta_{i,n}$, because there are zero segments only if the two endpoints are equal.

The Bayesian estimators of the original BPCR.
For the definition of the Bayesian estimators, we need to compute the evidence and the posterior distributions of the parameters. Since

$$\mathrm{L}_{k,n} = \mathrm{R}_{k,0} = \tbinom{n-1}{k-1} f(\boldsymbol{y}\,|\,k), \tag{S.9}$$

the *evidence* of the sample point $\boldsymbol{y}$ turns out to be

$$\mathrm{E} := f(\boldsymbol{y}) = \sum_{k=1}^{k_{\max}} f(\boldsymbol{y}\,|\,k) p_K(k) = \frac{1}{k_{\max}} \sum_{k=1}^{k_{\max}} \frac{\mathrm{L}_{k,n}}{\binom{n-1}{k-1}}. \tag{S.10}$$

Then, the posterior probabilities of the segment number and the boundaries can be written in the following way

$$\mathrm{C}_k \;:=\; p(k\,|\,\boldsymbol{y}) = \frac{f(\boldsymbol{y}\,|\,k)p(k)}{f(\boldsymbol{y})} = \frac{\mathrm{L}_{k,n}}{\mathrm{E}k_{\max}\binom{n-1}{k-1}} \qquad \forall k \in \mathbb{K} \tag{S.11}$$

$$\mathrm{B}_{ph} \;:=\; \mathrm{P}(T_p = h\,|\,\boldsymbol{y},\, k_0) = \frac{f(T_p = h,\, \boldsymbol{y}\,|\,K = k_0)}{f(\boldsymbol{y}\,|\,k_0)} = \frac{\mathrm{L}_{p,h}\mathrm{R}_{k_0-p,h}}{\mathrm{L}_{k_0,n}} \tag{S.12}$$

for each $p = 1, \ldots, k_0 - 1$ and for all $h \in \{p,\ \ldots,\ n-1\}$.

Now we can compute the estimators. The MAP (Maximum A Posteriori) estimate of the segment number given the sample point $\boldsymbol{y}$ is

$$\widehat{k} \;:=\; \arg\max_{k\in\mathbb{K}} p(k \mid \boldsymbol{y}) = \arg\max_{k\in\mathbb{K}} \frac{\mathrm{L}_{k,n}}{\binom{n-1}{k-1}}. \tag{S.13}$$

As we can see in Equation (S.12), the estimate for the boundaries needs the knowledge of the true number of segments, so we use $\hat{k}$ instead of k_0,

$$\widehat{t}_p := \arg\max_{h\in\{p,\,\ldots,\,n-(\widehat{k}-p)\}} \mathrm{P}(T_p = h \mid \boldsymbol{y}, \hat{k}) \qquad \text{for } p = 1,\ \ldots,\ \hat{k}-1. \tag{S.14}$$

The computation of the estimate of the rth moment of the level of the mth segment needs the knowledge of the segment number and the partition of the data (see Equation (S.4)), so we use the estimated ones

$$\widehat{\mu}_m^r := E[\mathrm{M}_m^r \mid \boldsymbol{y}, \widehat{\boldsymbol{t}}, \hat{k}] = \frac{\mathrm{A}_{i,j}^r}{\mathrm{A}_{i,j}^0}, \tag{S.15}$$

where $i = \widehat{t}_{m-1}$ and $j = \widehat{t}_m$, for all $m = 1,\ \ldots,\ \hat{k}$.

To estimate the segment level $\widetilde{\mathrm{M}}_s$ at a generic position s, we need at first to compute explicitly its posterior distribution:

$$f(\widetilde{\mu}_s \mid \boldsymbol{y}, K = k_0) \;=\; \sum_{m=1}^{k_0} \sum_{i=0}^{s-1} \sum_{j=s}^{n} f(\mu_m,\, T_{m-1} = i,\, T_m = j \mid \boldsymbol{y}, K = k_0) \tag{S.16}$$

$$\;=\; \frac{1}{\mathrm{L}_{k_0,n}} \sum_{m=1}^{k_0} \sum_{i=0}^{s-1} \sum_{j=s}^{n} \mathrm{L}_{m-1,i} f(\boldsymbol{y}_{i,j}, \mu_m \mid K_{i,j} = 1) \mathrm{R}_{k_0-m,j}. \tag{S.17}$$

Hence the estimate of $\widetilde{\mathrm{M}}_s$ given $\hat{k}$ is

$$\widehat{\widetilde{\mu}}_s := \sum_{i=0}^{s-1} \sum_{j=s}^{n} \mathrm{F}_{i,j}^0 \qquad \text{with } \mathrm{F}_{i,j}^0 := \frac{1}{\mathrm{L}_{\widehat{k},n}} \sum_{m=1}^{\widehat{k}} \mathrm{L}_{m-1,i} \mathrm{A}_{i,j}^0 \mathrm{R}_{\widehat{k}-m,j}, \tag{S.18}$$

for all $s = 1,\ \ldots,\ n$.

The boundary estimator $\widehat{\boldsymbol{T}}_{\text{joint}}$.

The boundary estimator $\widehat{\boldsymbol{T}}_{\text{joint}}$ is defined as

$$\widehat{\boldsymbol{T}}_{\text{joint}} \;:=\; \arg\max_{\boldsymbol{t}\in\mathbb{T}_{k_0,n}} p(\boldsymbol{t} \mid K = k_0, \boldsymbol{Y}). \tag{S.19}$$

The explicit formula for the joint boundary distribution, given k_0 and the sample point $\boldsymbol{y}$, is

$$p(\boldsymbol{t} \mid \boldsymbol{y},\, K = k_0) \;=\; \frac{\left[\prod_{p=0}^{k_0-1} f(\boldsymbol{y}_{t_p,t_{p+1}} \mid t_p,\, t_{p+1},\, K_{t_p,t_{p+1}} = 1)\right] p(\boldsymbol{t} \mid K = k_0)}{f(\boldsymbol{y} \mid K = k_0)}$$

$$\;=\; \frac{\left[\prod_{p=0}^{k_0-1} \mathrm{A}_{t_p,t_{p+1}}^0\right]}{\mathrm{L}_{k_0,n}}. \tag{S.20}$$

Thus, the estimated boundaries are

$$\widehat{\boldsymbol{t}}_{\text{joint}} = \operatorname*{arg\,max}_{\boldsymbol{t}\in\mathbb{T}_{k_0,n}} \prod_{p=0}^{k_0-1} \mathrm{A}^0_{t_p,t_{p+1}}. \tag{S.21}$$

This estimator can be computed by using the following recursion,

$$\begin{aligned} \mathrm{W}^1_{0,i} &:= \mathrm{A}^0_{0,i} \\ \mathrm{W}^{p+1}_{0,i} &:= \max_{h\in\{p,\dots,i-1\}} \mathrm{W}^p_{0,h}\mathrm{A}^0_{h,i} \qquad \text{for } p=1,\dots,\hat{k}-2, \end{aligned} \tag{S.22}$$

where $\mathrm{W}^p_{0,i}$ represents the maximum probability that $\boldsymbol{y}_{0,i}$ is divided into p segments over all combinations of the boundaries. As a consequence, the components of the vector of the boundary estimator turn out to be,

$$\widehat{t}_k = \operatorname*{arg\,max}_{h\in\{k,\dots,\widehat{t}_{k+1}-1\}} \mathrm{W}^k_{0,h}\mathrm{A}^0_{h,\widehat{t}_{k+1}} \qquad \text{for } k=\hat{k}-1,\dots,1, \tag{S.23}$$

with $\widehat{t}_{\hat{k}} := n$.

The boundary estimator $\widehat{\boldsymbol{T}}_{\text{BinErrAk}}$.
To compute the $\widehat{\boldsymbol{T}}_{\text{BinErrAk}}$ estimator, we need at first to make explicit the expected value in Equation (22), given the sample point $\boldsymbol{y}$,

$$\begin{aligned} \mathrm{E}\left[\sum_{i=1}^{n-1} \mathcal{T}_i\tau_i' \,\middle|\, \boldsymbol{y}\right] &= \sum_{k=2}^{k_{\max}} \sum_{\boldsymbol{\tau}\in\mathcal{T}_{k,n}} \sum_{i=1}^{n-1} \tau_i\tau_i' p(\boldsymbol{\tau}\,|\,\boldsymbol{y},\,k)p(k\,|\,\boldsymbol{y}) \\ &= \sum_{i=1}^{n-1} \delta_{\tau_i',1} \sum_{k=2}^{k_{\max}} \sum_{p=1}^{\min(i,k-1)} \frac{\mathrm{L}_{p,i}\mathrm{R}_{k-p,i}}{\mathrm{L}_{k,n}} \frac{\mathrm{L}_{k,n}}{\binom{n-1}{k-1} k_{\max}\mathrm{E}} && \text{(S.24)} \\ &= \sum_{\{i=1,\dots,n-1\,:\,\tau_i'=1\}} \mathrm{P}(\tau_i = 1\,|\,\boldsymbol{Y}=\boldsymbol{y}), && \text{(S.25)} \end{aligned}$$

where

$$\begin{aligned} \mathrm{P}(\tau_i=1\,|\,\boldsymbol{Y}=\boldsymbol{y}) &= \sum_{k=2}^{k_{\max}} \mathrm{P}(\boldsymbol{t}\in\mathbb{T}_{k,n} : \exists p \text{ such that } t_p = i\,|\,\boldsymbol{y},\,k)p(k\,|\,\boldsymbol{y}) \\ &= \sum_{k=2}^{k_{\max}} \sum_{p=1}^{\min(i,k-1)} \mathrm{P}(\boldsymbol{t}\in\mathbb{T}_{k,n} : t_p = i\,|\,\boldsymbol{y},\,k)p(k\,|\,\boldsymbol{y}). \end{aligned} \tag{S.26}$$

Hence, to find the estimated $\widehat{\boldsymbol{\mathcal{T}}}_{\text{BinErrAk}}$, we need to find the $\widehat{k}-1$ highest $\{\mathrm{P}(\tau_i = 1\,|\,\boldsymbol{Y} = \boldsymbol{y})\}_{i=1}^{n-1}$ (corresponding to the indices $i_1,\,\dots,\,i_{\widehat{k}-1}$) and then take $\widehat{\boldsymbol{\tau}}_{\text{BinErrAk}}$ such that $\widehat{\tau}_{i_p} = 1$ for $p = 1,\,\dots,\,\widehat{k}-1$. Using the inverse function of (19), we obtain the corresponding value of the estimator $\widehat{\boldsymbol{T}}_{\text{BinErrAk}}$.

The BRCAk.
The computation of the BRCAk is simply given by

$$\begin{aligned}\widehat{\widetilde{\mathrm{M}}}_s &:= E[\widetilde{\mathrm{M}}_s \,|\, \boldsymbol{y}] \\ &= \sum_{k=1}^{k_{\max}} p(k \,|\, \boldsymbol{y}) \int_{\mathbb{R}} \widetilde{\mu}_s f(\widetilde{\mu}_s \,|\, \boldsymbol{y}, k) d\widetilde{\mu}_s \\ &= \sum_{k=1}^{k_{\max}} \mathrm{C_k} \sum_{i<t\leq j} \mathrm{F}^0_{i,j}(k), \qquad \text{(S.27)}\end{aligned}$$

where in the last equation we used the notations of Hutter [11,12] with the only difference that now $\mathrm{F}^0_{i,j}$ must be computed for all possible k and so we need to make explicit this dependency. Unfortunately, the computation of this quantity required time $O(n^2 k_{\max}^2)$, hence it could be a problem with samples of big size.

S.2 Some error measure definitions

Definition of the MSE measure.
The mean square error is defined as the expected value of the square error and so it is estimated with the corresponding arithmetic mean. Hence, given N estimated values $\hat{\theta}_1, \ldots, \hat{\theta}_N$ of a generic parameter θ, the estimated Mean Square Error of θ is defined as

$$\mathrm{MSE}(\theta) := \frac{1}{N}\sum_{i=1}^{N}(\hat{\theta}_i - \theta)^2.$$

Definition of the mean relative error.
Given N estimated values $\hat{\theta}_1, \ldots, \hat{\theta}_N$ of a generic parameter θ, the estimated mean relative error over all datasets is

$$\frac{\Delta\hat{\theta}}{\theta} := \frac{1}{mn}\sum_{j=1}^{m}\sum_{i=1}^{n}\frac{\left|\hat{\theta}_{ij} - \theta_j\right|}{\theta_j},$$

where m is the number of datasets, n the number of samples in each dataset and θ_{ij} is the estimate of θ_j based on the ith sample in the jth dataset.

S.3 Supplementary Tables

$\Delta\hat{\nu}/\nu$	0.6376
$\Delta\hat{\sigma}^2/\sigma^2$	0.1524
$\Delta\hat{\rho}^2/\rho^2$	8.6217
$\Delta\hat{\rho}_1^2/\rho_1^2$	0.5840

Table S.1: Estimated mean relative error of the estimators $\hat{\nu}$, $\hat{\sigma}^2$, $\hat{\rho}^2$ and $\hat{\rho}_1^2$ over all the datasets used. The results show that $\hat{\sigma}^2$ had the lowest error. The error of $\hat{\nu}$ was high but acceptable. The estimator $\hat{\rho}_1^2$ was significantly better than $\hat{\rho}^2$ with respect to this error measure.

error \ $\widehat{T}$	$\widehat{\boldsymbol{T}}_{01}$	$\widehat{\boldsymbol{T}}_{joint}$	$\widehat{\boldsymbol{T}}_{BinErr}$	$\widehat{\boldsymbol{T}}_{BinErrAk}$
sum 0-1	14.37 ± 0.176	16.04 ± 0.195	16 ± 0.194	15.99 ± 0.193
joint 0-1	0.9393 ± 0.00436	0.945 ± 0.0042	0.9387 ± 0.00438	0.9387 ± 0.00438
binary	10.48 ± 0.126	8.3787 ± 0.0961	7.7433 ± 0.09	7.7793 ± 0.0903
aver. square	35.12 ± 15.667	67.3 ± 17.036	37.85 ± 15.792	38.76 ± 15.824

Table S.2: Estimated mean errors $\pm$ standard deviation of the estimators of $\boldsymbol{t}^0$ applied on a dataset without replicates with $\sigma^2 = 0.1$ and $\rho^2 = 0.5$, using k_0, $\hat{\nu}$, $\hat{\sigma}^2$ and $\hat{\rho}_1^2$. The estimator $\widehat{\boldsymbol{T}}_{01}$ minimized the sum 0-1 error and the average square error. The errors of $\widehat{\boldsymbol{T}}_{BinErr}$ and $\widehat{\boldsymbol{T}}_{BinErrAk}$ were always very close and low.

gene region	FISH CN	mBPCR $\hat{\rho}^2$	mBPCR $\hat{\rho}_1^2$	CBS	CGHseg	HMM	GLAD	BioHMM	Rendersome
BCL6	2/3	2.89	2.85	2.89	2.90	2.05	2.79	2.85	2.86
C-MYC	2	2.02	1.99	2.06	2.12	2.05	2.07	2.02	2.02
CCND1	2	2.01	1.92	2.01	2.05	2.05	2.07	2.02	2.02
BIRC3	2/3	2.01	2.22	2.01	2.05	2.05	2.07	2.02	2.02
ATM	2	2.01	1.80	2.01	2.05	2.05	2.07	2.02	2.02
D13S319	2	2.03	2.40	1.98	2.03	2.05	2.01	2.02	*2.89*
LAMP1	2	1.82	1.76	1.98	1.98	2.05	2.01	2.02	2.02
TP53	1/2	1.11	1.17	1.11	1.11	1.10	1.55	1.10	1.20
MALT1	2/3	2.12	2.25	2.02	2.12	2.05	2.09	2.02	2.02
BCL2	2	2.12	2.25	2.02	2.12	2.05	2.09	2.02	2.02

Table S.3: Copy number estimation results obtained on sample REC-1. Globally, all methods behaved equally well on this data. Only Rendersome was unable to detect the correct copy number of *D13S319*.

gene region	FISH CN	mBPCR $\hat{\rho}^2$	mBPCR $\hat{\rho}_1^2$	CBS	CGHseg	HMM	GLAD	BioHMM	Renders.
BCL6	2	2.11	2.10	2.11	2.07	2.11	1.85	2.12	2.04
C-MYC	2	2.07	2.00	2.08	2.11	1.99	*6.22/1.37*	2.12	2.04
CCND1	2	2.06	2.03	2.06	2.34	2.20	2.4	2.12	2.04
BIRC3	2/3	2.06	1.76	2.06	*1.14/2.34*	2.11	2.4	2.12	2.04
ATM	1	*2.06*	*2.01/1.61*	*2.06*	*2.34*	*2.11/1.14*	*2.4*	*2.12*	*2.04*
D13S319	2	2.01	2.00	2.03	2.05	2.07	2.26	2.12	2.04
LAMP1	2	2.01	2.00	2.03	2.05	1.98	1.58	2.12	2.04
TP53	1	1.10	1.16	1.13	1.01	1.13	*1.85*	1.36	1.08
MALT1	3	3.36	3.05	3.17	3.04	*5.30*	*2.16*	*4.78*	*4.28*
BCL2	ampl	5.46	5.10	5.52	6.12	5.30	*2.16*	4.78	7.22

Table S.4: Copy number estimation results obtained on sample GRANTA-519. On this data, the GLAD method often did not detect the right gene copy number. The method mBPCR with $\hat{\rho}_1^2$ estimated the gene copy number always well, apart from *ATM* whose copy number was estimated different from the FISH copy number by all methods.

gene region	FISH CN	mBPCR $\hat{\rho}^2$	mBPCR $\hat{\rho}_1^2$	CBS	CGHseg	HMM	GLAD	BioHMM	Rendersome
BCL6	3/2	3.06	3.06	3.02	3.06	2.96	2.98	3.04	2.98
C-MYC	ampl	7.12	7.10	7.14	6.87	*6.70/2.63*	7.28	6.51	7.72
CCND1	2	*3.51*	*3.51*	*3.51*	*3.51*	*3.44*	*3.51*	*3.52*	*3.60*
BIRC3	4/5	4.20	4.19	4.20	4.24	4.24	4.23	4.26	3.60
ATM	4	4.20	4.19	4.20	4.24	4.24	4.23	4.26	3.60
D13S319	4	3.72	3.72	3.81	3.82	3.72	3.81	3.73	3.64
LAMP1	4	3.67	3.67	3.82	3.67	3.72	3.69	3.73	3.64
TP53	2/3	2.57	2.69	2.22	2.76	2.34	2.76	2.83	2.90
MALT1	4	3.52	3.52	3.59	3.59	3.50	3.55	3.52	3.50
BCL2	4	3.52	3.52	3.59	3.59	3.50	3.55	3.52	3.50

Table S.5: Copy number estimation results obtained on 250K Array data of sample JEKO-1. All methods behaved equally good. The method HMM had problem in determining the right position of one breakpoint around the *C-MYC* amplification. All methods estimated the copy number of *CCND1* differently from FISH technique.

S.4 Supplementary Figures

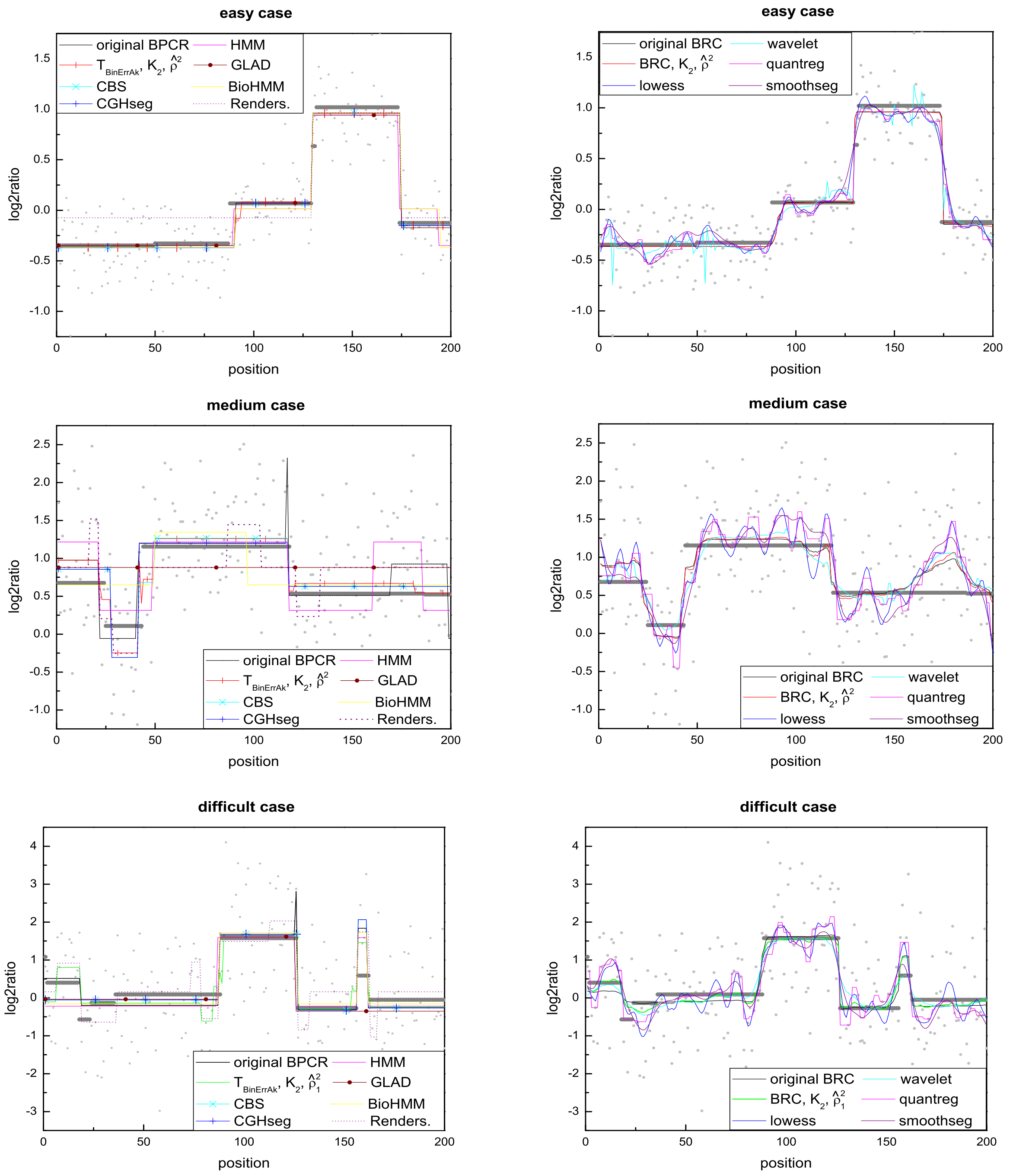

Figure S.1: Estimated profiles of the data shown in Figure 1, obtained by applying several methods. In each plot, the grey segments represent the true profile and the dots are the raw data points.

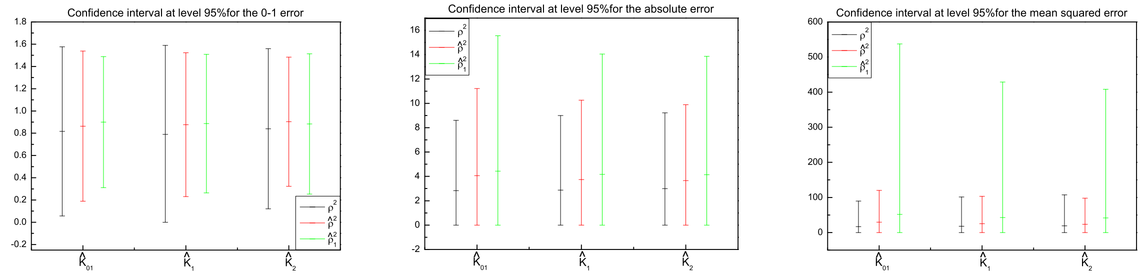

Figure S.2: Confidence intervals at 95% of the estimators of k_0 for, respectively, the 0-1 error, the absolute error and the squared error obtained on a dataset without replicates with $\sigma^2 = 0.1$ and $\rho^2 = 0.5$. The graphs show that, in each situation, $\widehat{K}_2$ always had the lowest upper bound of the interval. Using $\hat{\rho}^2$ the confidence intervals were shorter.

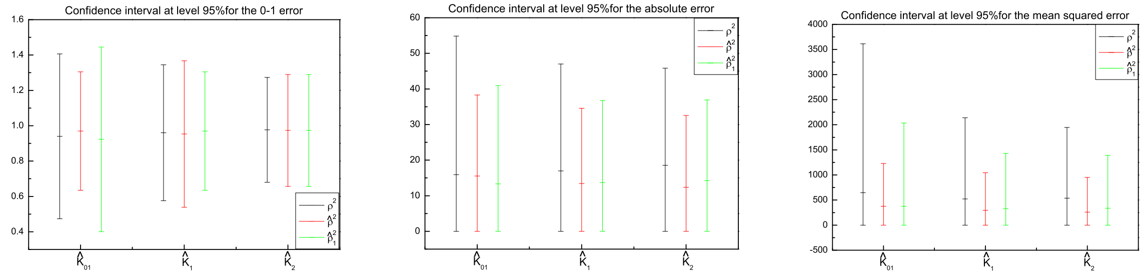

Figure S.3: Confidence intervals at 95% of the estimators of k_0 for, respectively, the 0-1 error, the absolute error and the squared error obtained on a dataset without replicates with $\sigma^2 = 0.3$ and $\rho^2 = 0.05$. The graphs show that, in each situation, $\widehat{K}_2$ always had the lowest upper bound of the interval.

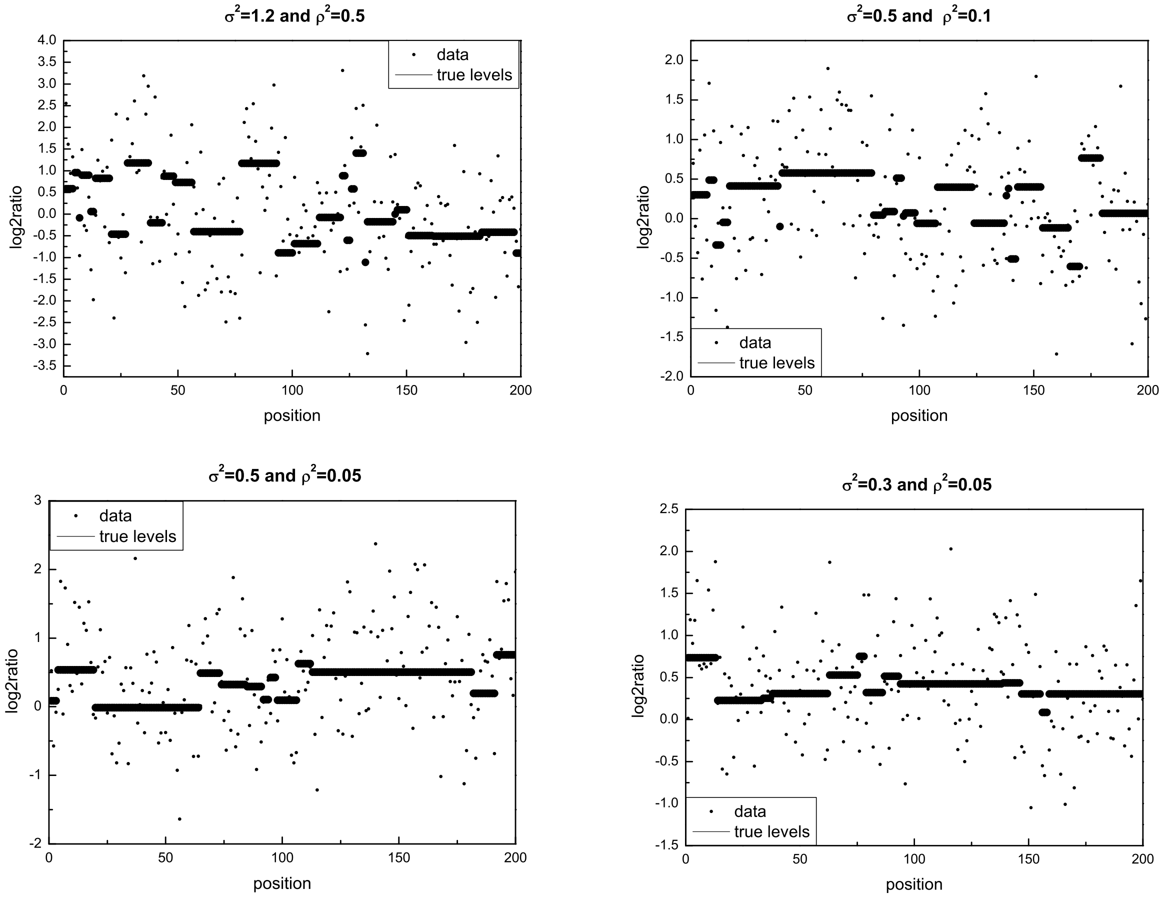

Figure S.4: True profiles of some datasets with replicates used in the comparisons.

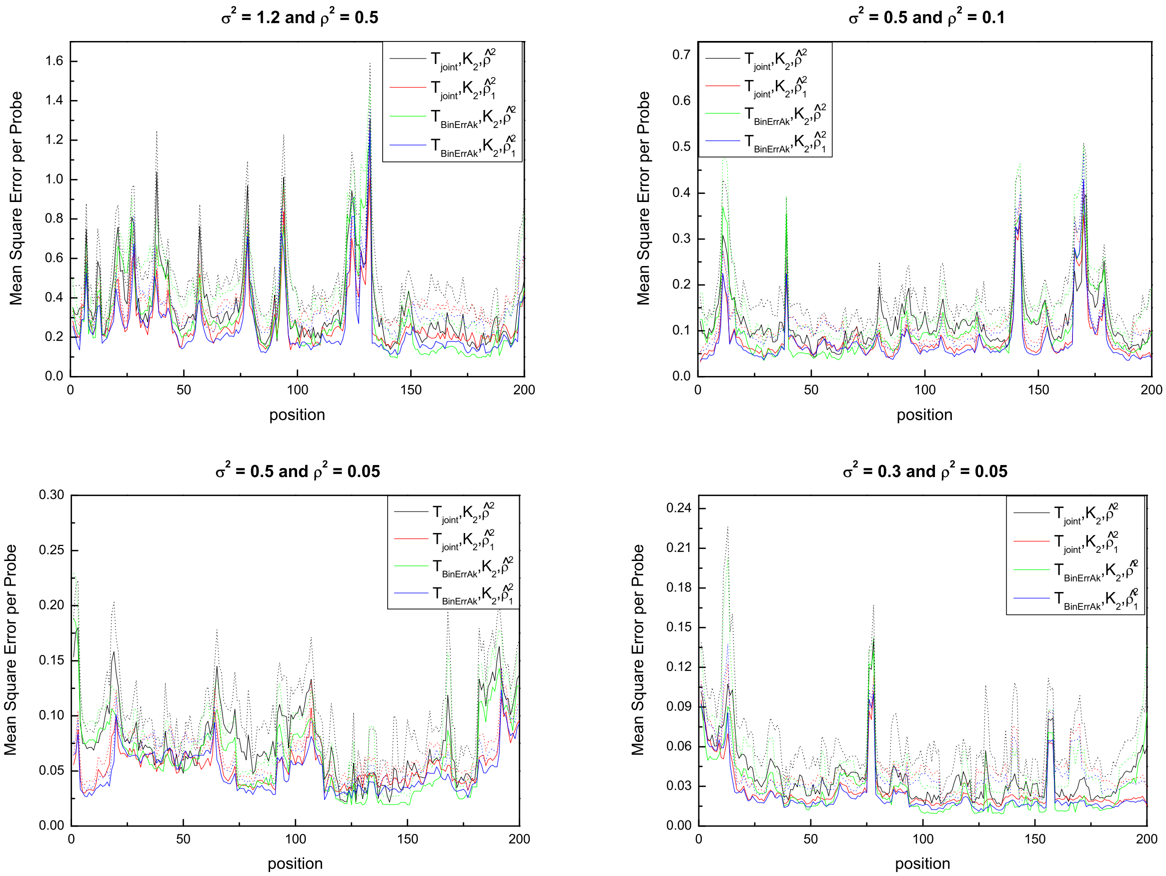

Figure S.5: Comparison of the segment level estimation by using $\widehat{\boldsymbol{T}}_{joint}$ or $\widehat{\boldsymbol{T}}_{BinErrAk}$ with the different ρ^2 estimators, on four datasets with replicates. The corresponding true profiles are in Figure S.4. In general, using $\widehat{\boldsymbol{T}}_{BinErrAk}$ we obtained a lower MSE per probe than using $\widehat{\boldsymbol{T}}_{joint}$. For a fixed boundary estimator, often the error was lower by using $\hat{\rho}_1^2$ on these datasets where $\sigma^2 \gg \rho^2$.

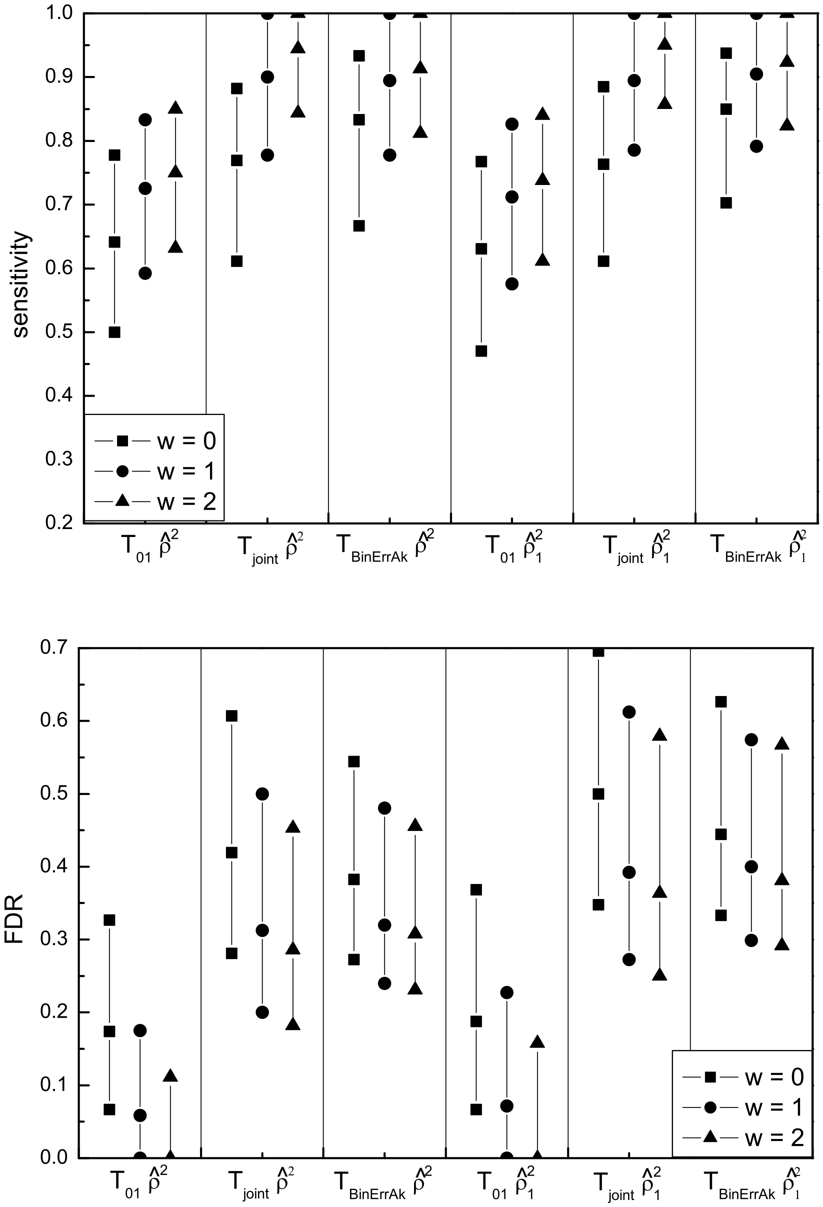

Figure S.6: Comparison of the sensitivity and FDR computed on the results obtained on dataset *Simulated Chromosomes* using all the piecewise constant methods. Using $\widehat{\rho}^2$ instead of $\widehat{\rho}_1^2$, the FDR was lower. The estimator $\widehat{\boldsymbol{T}}_{BinErrAk}$ had the highest sensitivity and the second lowest FDR. The FDR of $\widehat{\boldsymbol{T}}_{01}$ was the lowest, but this is due to the fact that it reduces the number of the estimated breakpoints by assigning the same position to different breakpoints. In conclusion, $\widehat{\boldsymbol{T}}_{BinErrAk}$ with $\widehat{\rho}^2$ was the best performing estimator on this dataset.

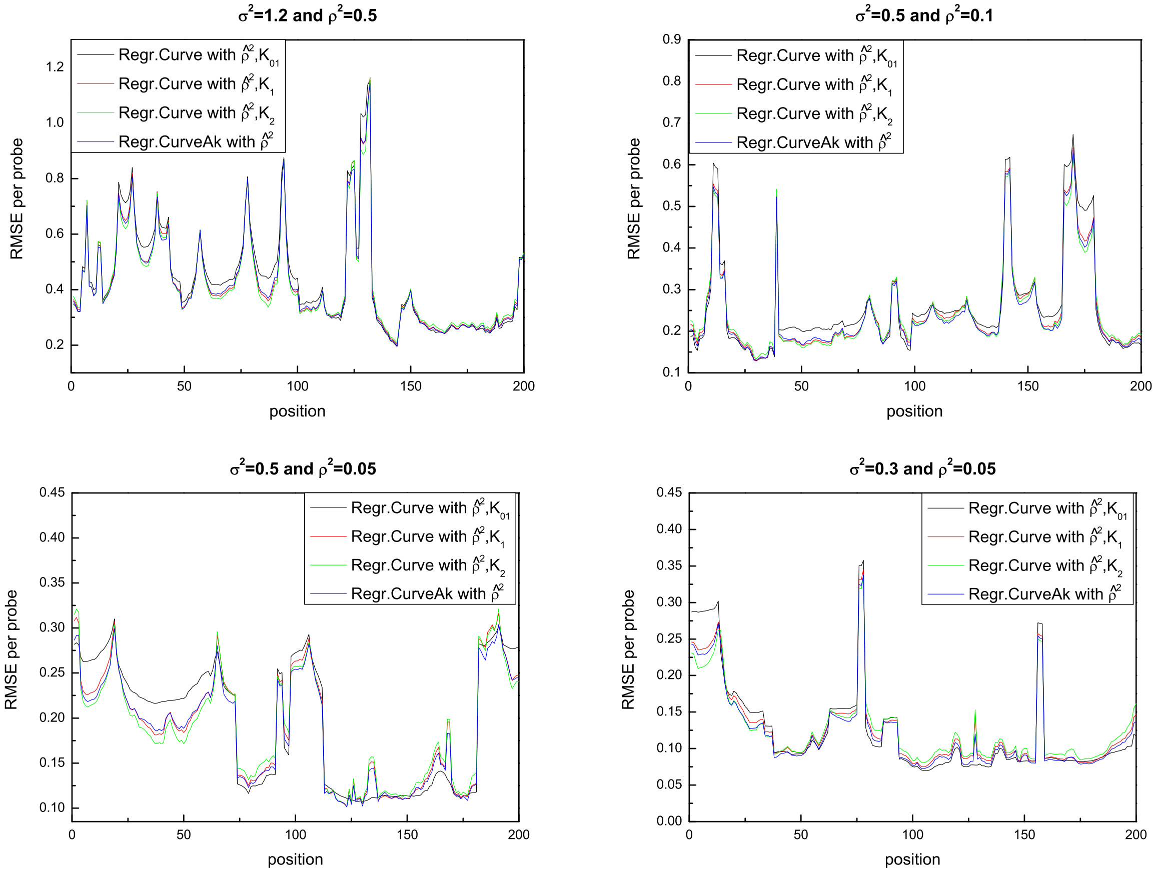

Figure S.7: RMSE per probe of the several Bayesian regression curves, using $\hat{\rho}^2$ as the estimator of ρ^2, on four datasets with replicates. The corresponding true profiles are in Figure S.4. The graphs show that, using $\hat{\rho}^2$, BRCAk always had the lowest RMSE per probe and thus performed better than the other BRCs.

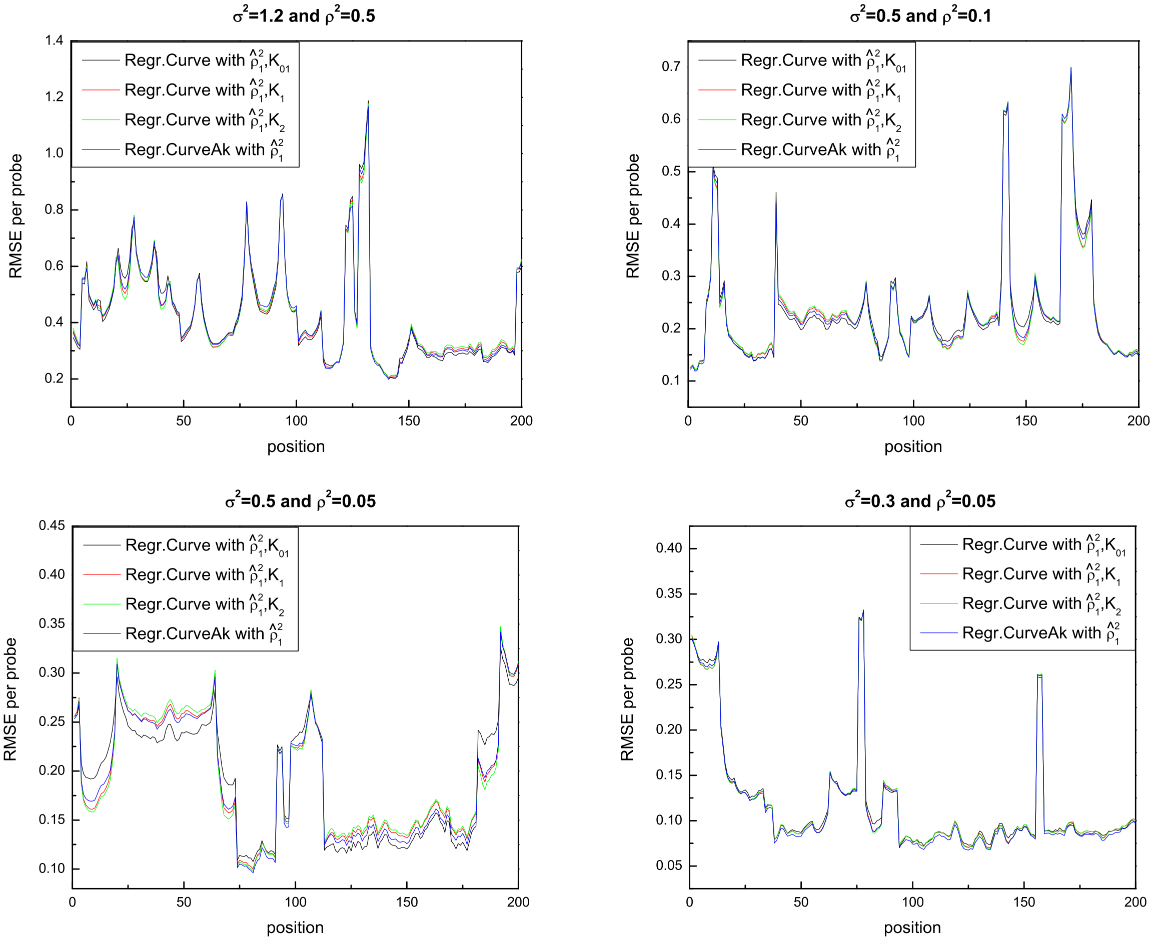

Figure S.8: RMSE per probe of the several Bayesian regression curves, using $\hat{\rho}_1^2$ as the estimator of ρ^2, on four datasets with replicates. The corresponding true profiles are in Figure S.4. Using the estimator $\hat{\rho}_1^2$, all the regression curves gave similar RMSE per probe curve.

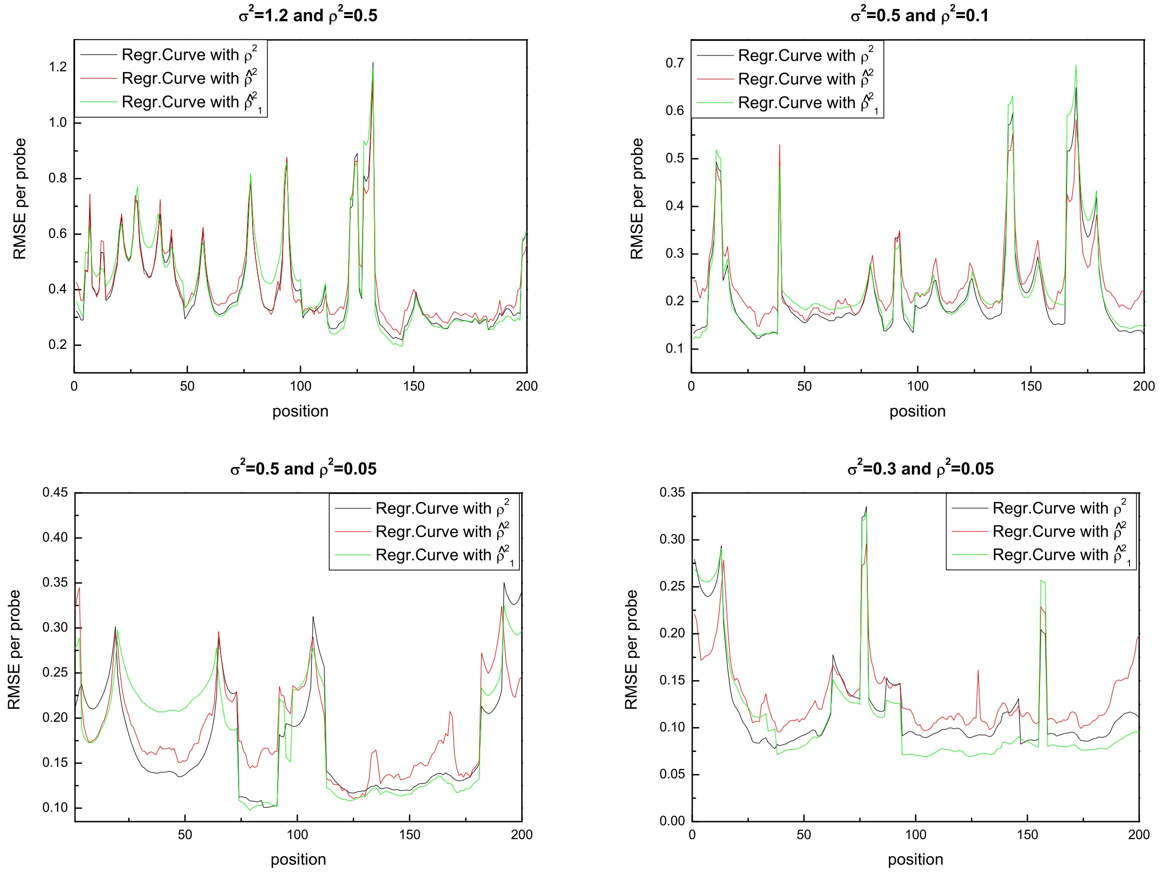

Figure S.9: RMSE per probe of the Bayesian regression curve using different estimators of ρ^2, on four datasets with replicates. The corresponding true profiles are in Figure S.4. The graphs do not show clearly which ρ^2 estimator was better with respect to this error measure. Sometimes the error committed using $\hat{\rho}_1^2$ was lower than using $\hat{\rho}^2$, probably because $\hat{\rho}_1^2$ can lead to a slight overfitting.

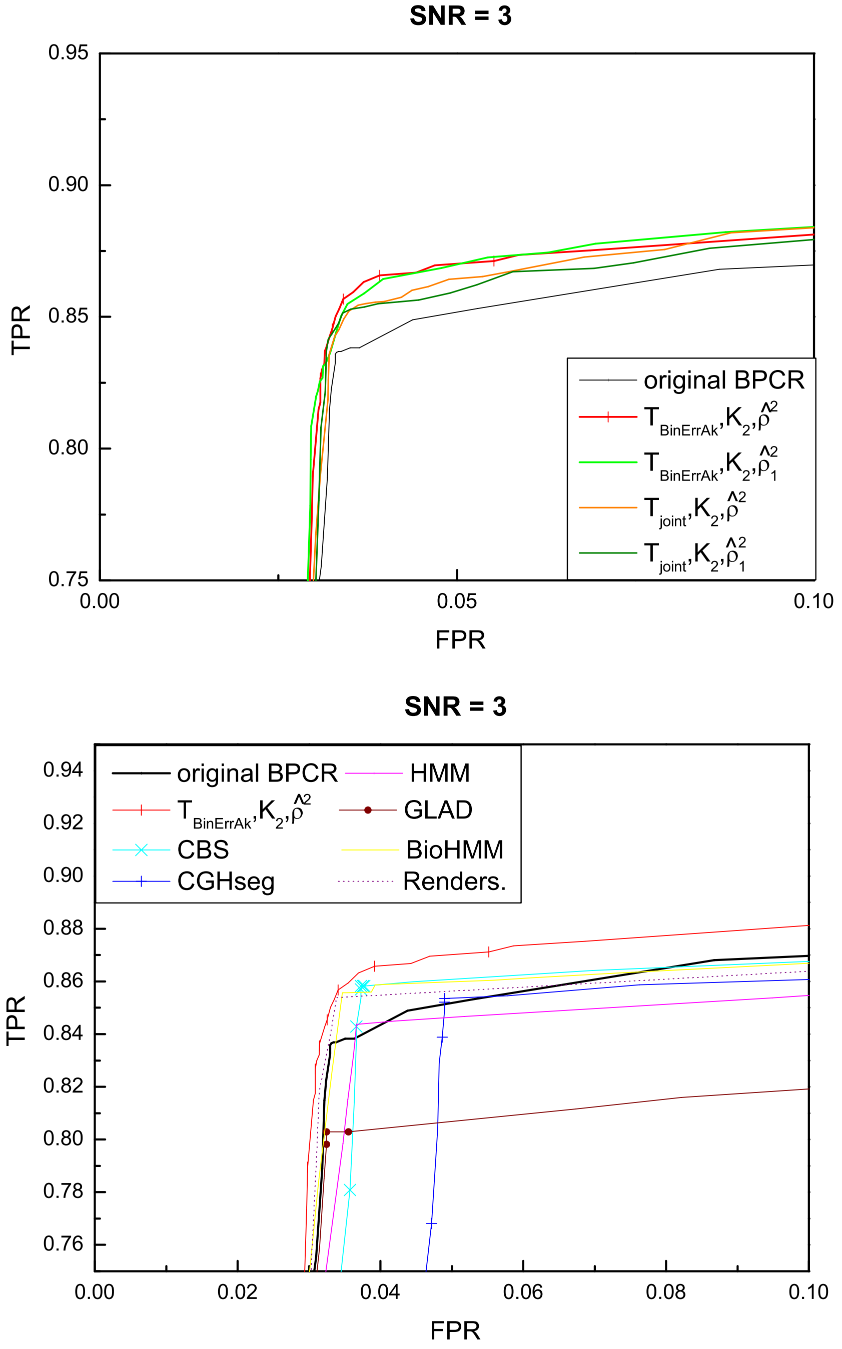

Figure S.10: Zoomed ROC curves of several piecewise constant methods applied to dataset with SNR = 3. On this easy type of data, all the methods (apart from GLAD) performed well, since their ROC curves were close to the top left corner of the plot.

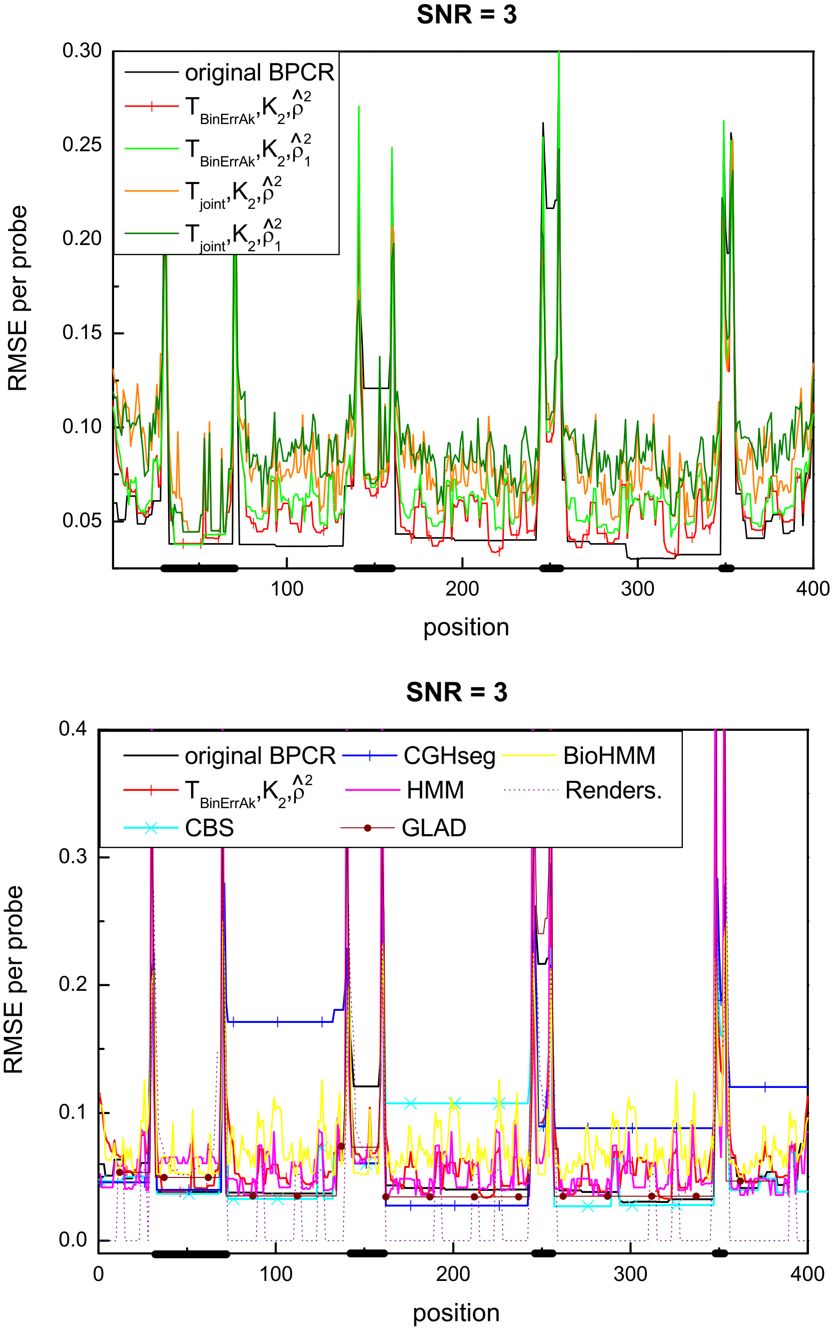

Figure S.11: RMSE of several piecewise constant methods applied to dataset with SNR = 3. The black segments on the horizontal axis correspond to the regions of aberration. On this dataset, HMM and the modified BPCR with $\hat{\rho}^2$, $\widehat{K}_2$ and $\widehat{\boldsymbol{T}}_{BinErrAk}$ achieved a low RMSE both inside and outside the aberrations, compared to the other algorithms. Nevertheless, HMM had the highest error at the breakpoint positions. Hence, the modified BPCR with $\hat{\rho}^2$, $\widehat{K}_2$ and $\widehat{\boldsymbol{T}}_{BinErrAk}$ performed better than all other methods with respect to the RMSE per probe measure.

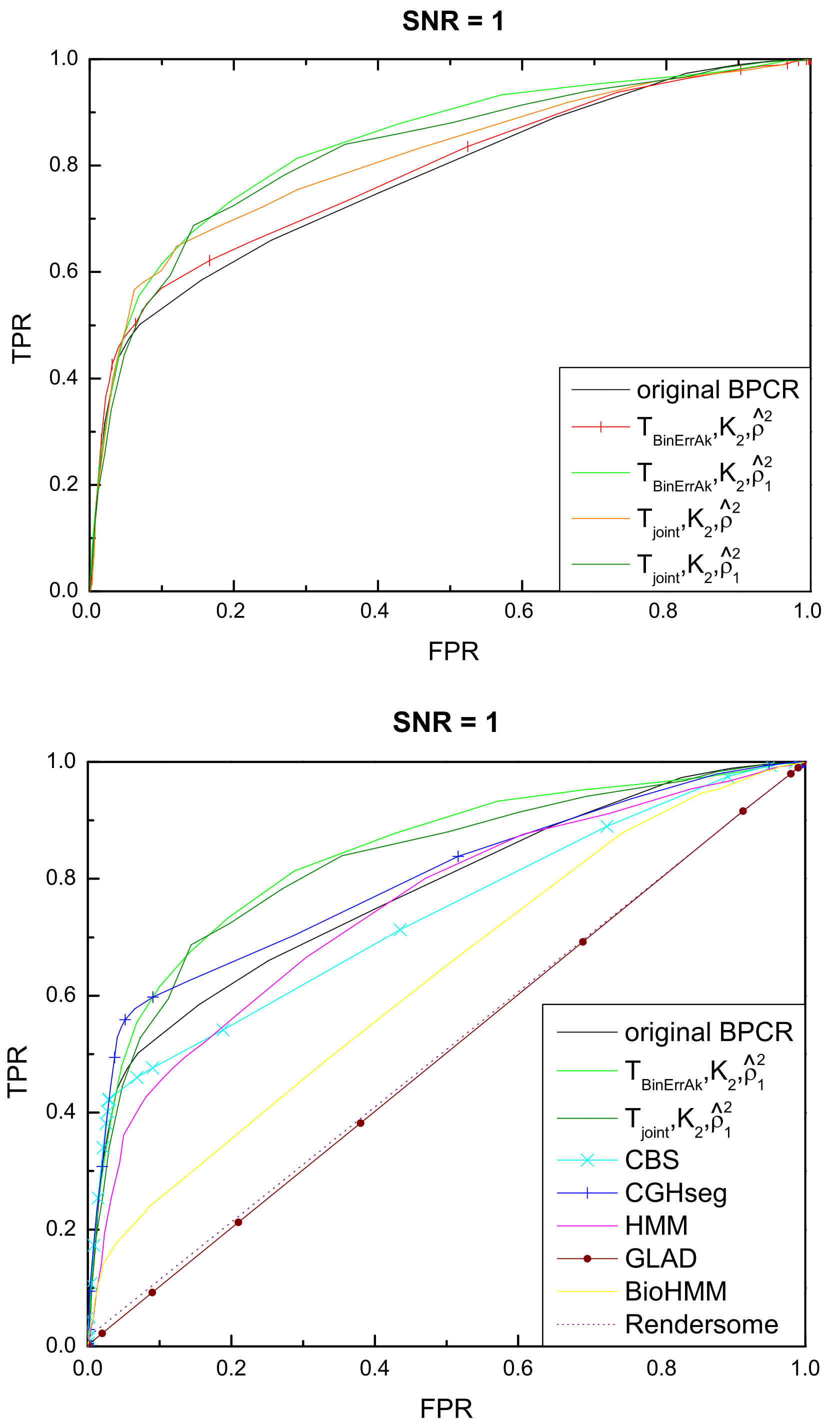

Figure S.12: ROC curves of several piecewise constant methods applied to dataset with SNR = 1. On this type of dataset with high noise, the modified BPCR with $\hat{\rho}_1^2$, $\widehat{K}_2$ and $\widehat{\boldsymbol{T}}_{BinErrAk}$ was the best performing method with respect to the ROC curve measure, because its curve was globally the highest one at the top left corner of the plot. GLAD, Rendersome and BioHMM were the worse methods in detecting the aberrations on this dataset.

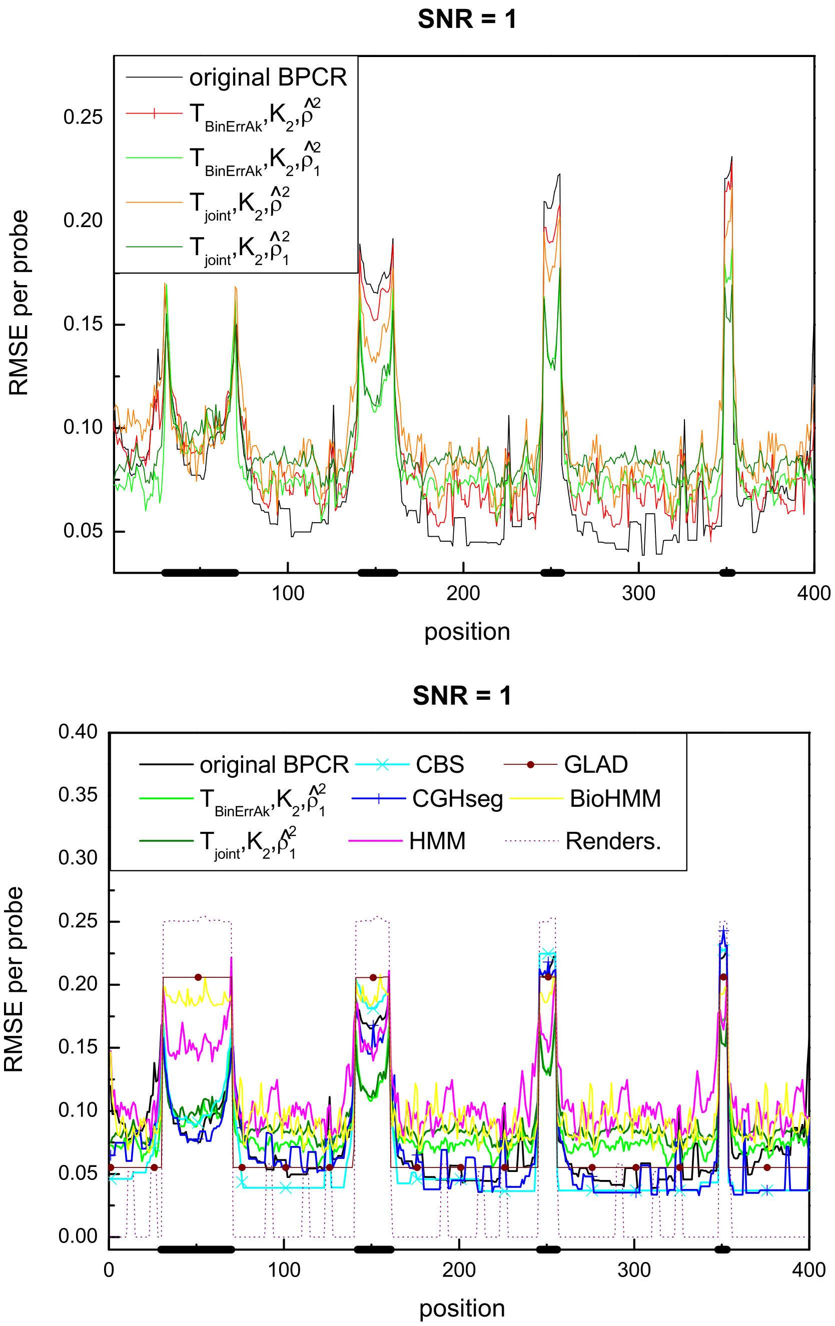

Figure S.13: RMSE of several piecewise constant methods applied to dataset with SNR = 1. The black segments on the horizontal axis correspond to the regions of aberration. On this very noisy dataset, the modified BPCR with $\hat{\rho}_1^2$, $\widehat{K}_2$ and $\widehat{\boldsymbol{T}}_{BinErrAk}$ always had a low RMSE per probe, even though its error was not the lowest one outside the aberrations and inside the first one. On the contrary, CBS and CGHseg had the lowest error in these regions, but the highest error inside the small aberrations. Hence, globally the modified BPCR with $\hat{\rho}_1^2$, $\widehat{K}_2$ and $\widehat{\boldsymbol{T}}_{BinErrAk}$ performed better than the other algorithms on this dataset, with respect to the RMSE measure.

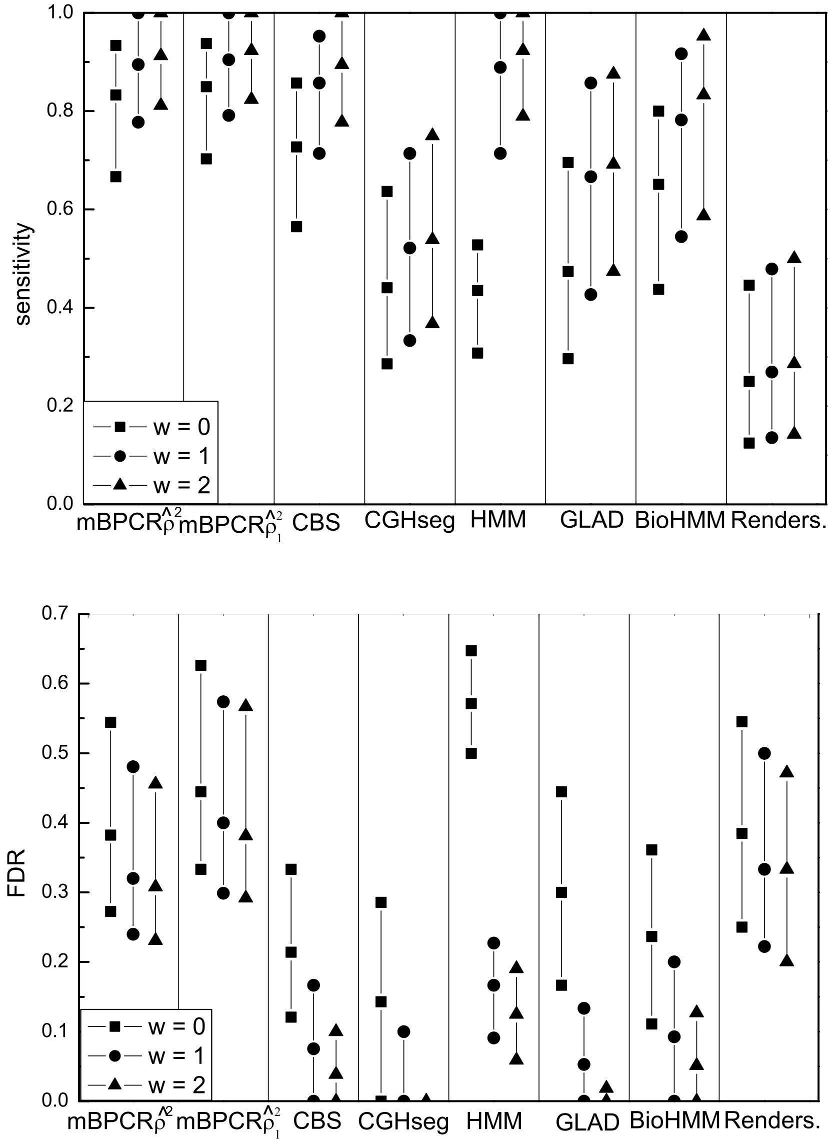

Figure S.14: Comparison of the sensitivity and FDR computed on the results obtained on dataset *Simulated Chromosomes*, using several piecewise constant methods. The mBPCR method (considering both ρ^2 estimators) had the highest sensitivity, hence it was the method that determined the breakpoints location with highest precision. Nevertheless, it had a higher FDR than CBS.

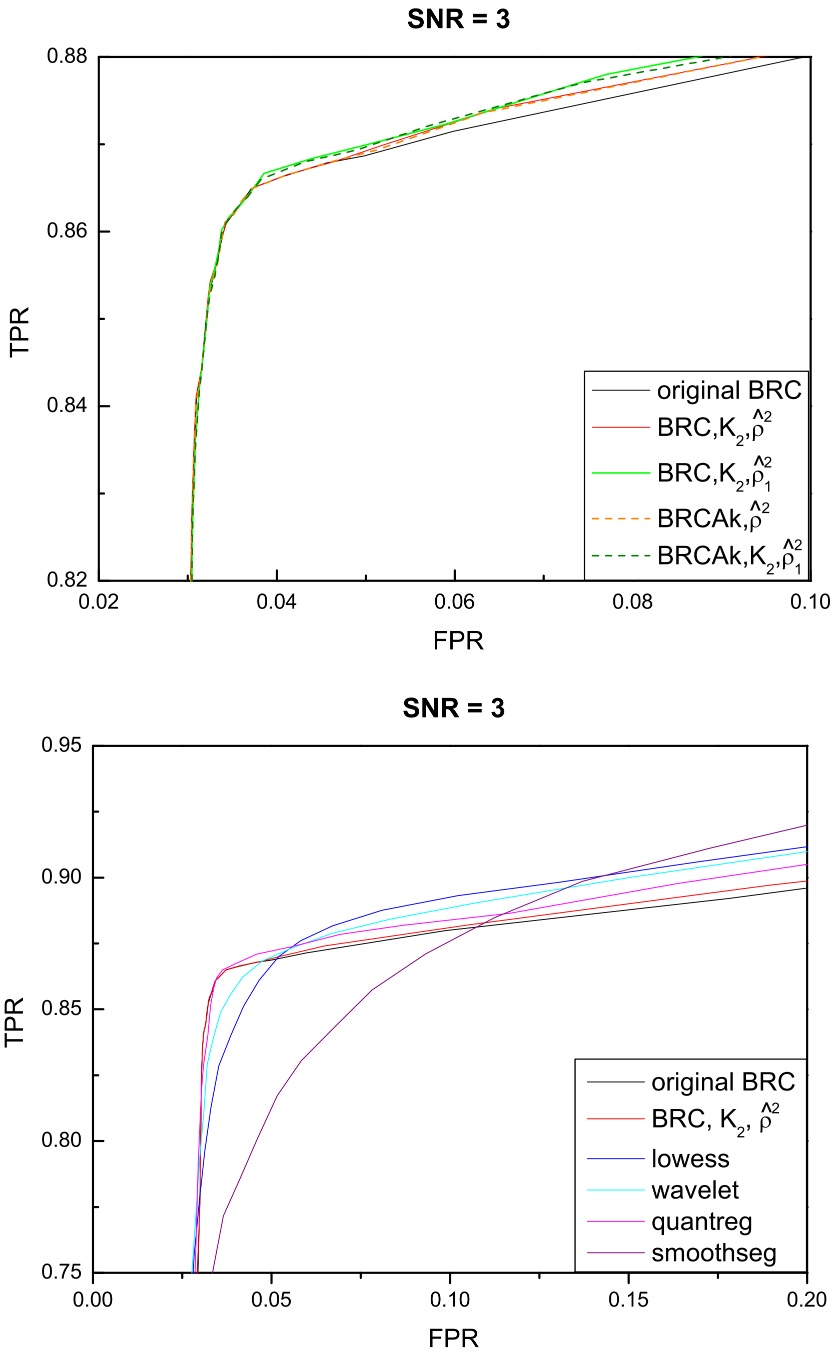

Figure S.15: Zoomed ROC curves of several smoothing methods applied to dataset with SNR = 3. The intersection among the ROC curves was due to the differences of the methods in the level estimation outside the aberrations. The more oscillating were the estimated curves in these regions, the closer were the corresponding ROC curves to the top side of the graph. In our case, an oscillating estimated profile is very different from the true one.

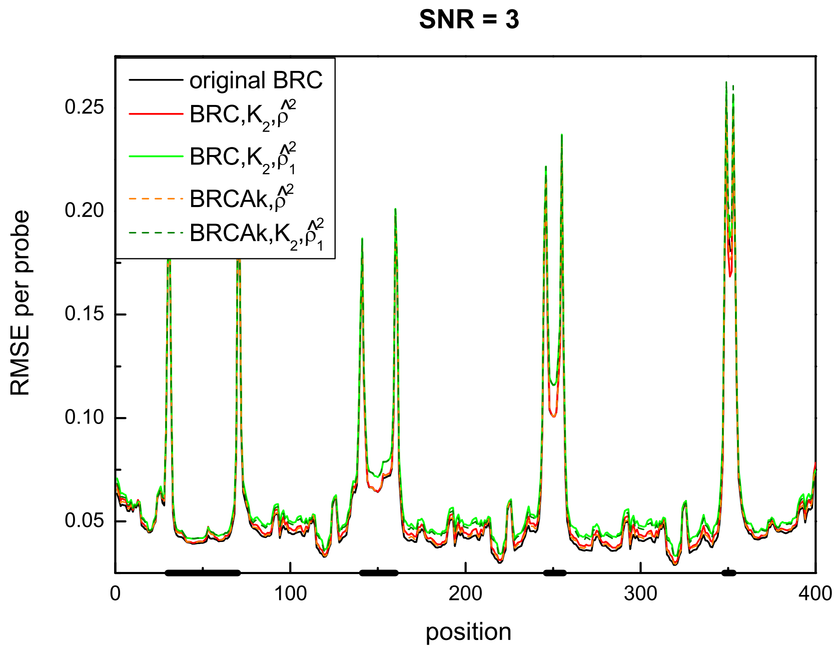

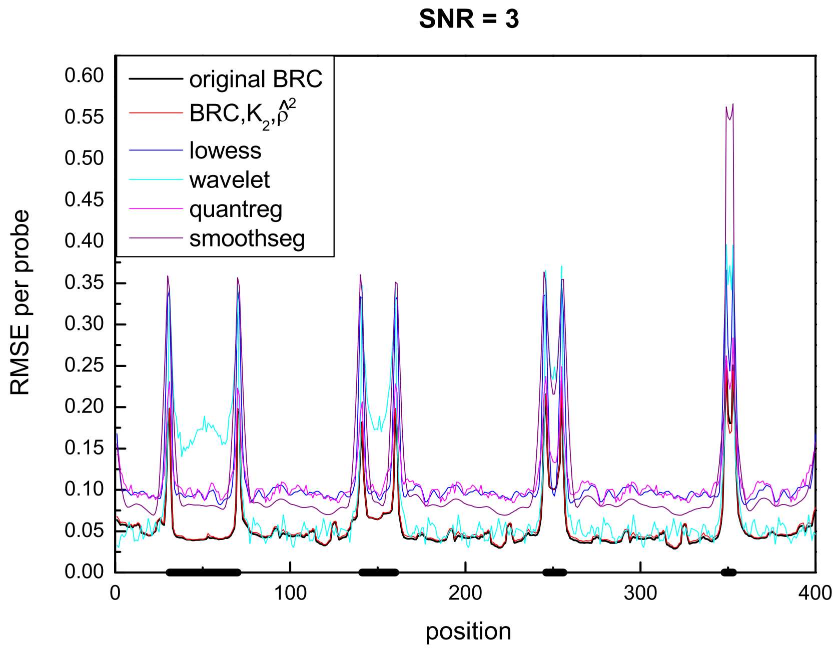

Figure S.16: RMSE of several smoothing methods applied to dataset with SNR = 3. The black segments on the horizontal axis correspond to the regions of aberration. On this dataset, both the original BRC and the version of BRC with $\widehat{K}_2$ and $\widehat{\rho}^2$ had everywhere the lowest error.

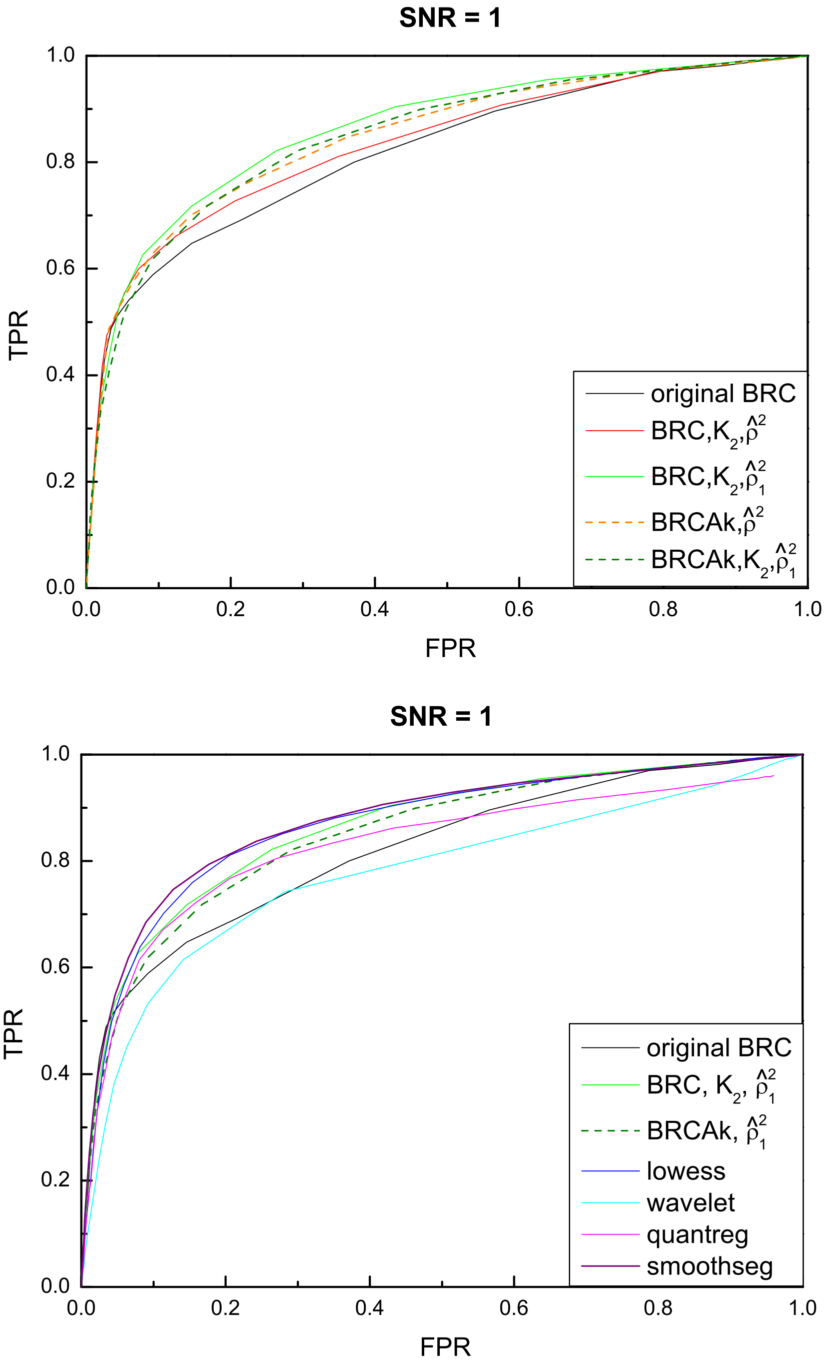

Figure S.17: ROC curves of several smoothing methods applied to dataset with SNR = 1. On this very noisy data, the methods smoothseg and lowess seemed to be the best ones, since their ROC curves were the highest at the top left corner of the plot. The third best method was BRC with $\widehat{K}_2$ and $\widehat{\rho}_1^2$.

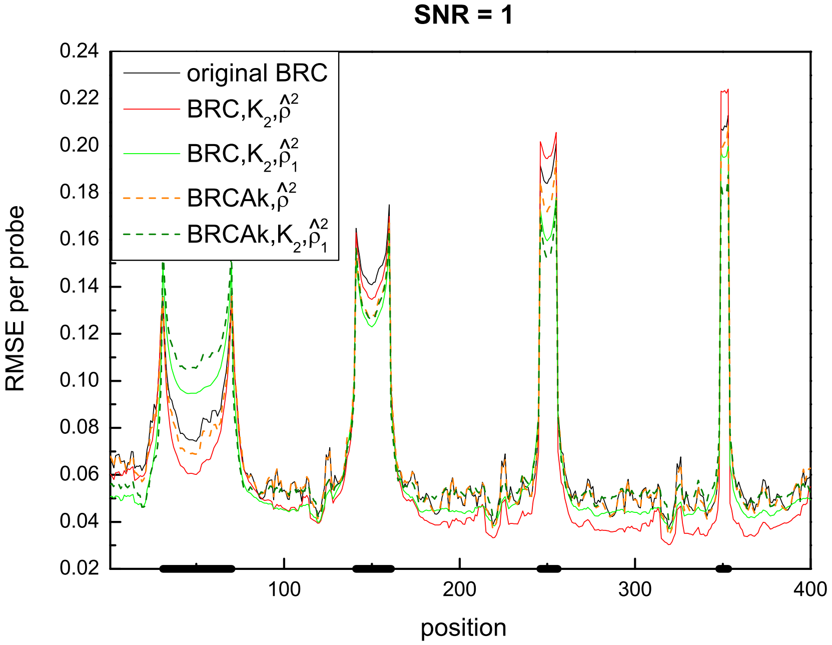

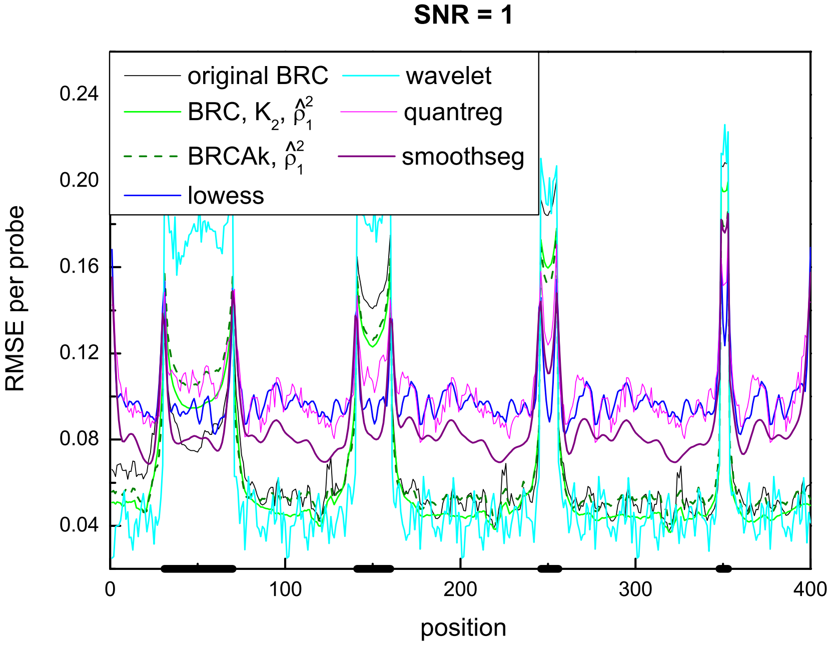

Figure S.18: RMSE of several smoothing methods applied to dataset with SNR = 1. The black segments on the horizontal axis correspond to the regions of aberration. The graphs show that the method lowess, quantreg and smoothseg had more or less the same error inside and outside the aberrations. Instead, the BRC version with $\widehat{K}_2$ and $\widehat{\rho}_1^2$ and BRCAk with $\widehat{\rho}_1^2$ had a very low error outside the aberrations and not the highest error inside them, thus globally they performed better than the other algorithms with respect to the RMSE measure.
